# Description of grain weight distribution leading to genomic selection for grain-filling characteristics in rice

**DOI:** 10.1371/journal.pone.0207627

**Published:** 2018-11-20

**Authors:** Shiori Yabe, Hiroe Yoshida, Hiromi Kajiya-Kanegae, Masanori Yamasaki, Hiroyoshi Iwata, Kaworu Ebana, Takeshi Hayashi, Hiroshi Nakagawa

**Affiliations:** 1 Institute of Crop Science, NARO, Tsukuba, Ibaraki, Japan; 2 PRESTO, JST, Kawaguchi, Saitama, Japan; 3 Institute for Agro-Environmental Sciences, NARO, Tsukuba, Ibaraki, Japan; 4 Department of Agricultural and Environmental Biology, Graduate School of Agricultural and Life Science, The University of Tokyo, Tokyo, Japan; 5 Food Resources Education and Research Center, Graduate School of Agricultural Science, Kobe University, Kasai, Hyogo, Japan; 6 Genetic Resources Center, NARO, Tsukuba, Ibaraki, Japan; Murdoch University, AUSTRALIA

## Abstract

Grain-filling ability is one of the factors that controls grain yield in rice (*Oryza sativa* L.). We developed a method for describing grain weight distribution, which is the probability density function of single grain weight in a panicle, using 128 Japanese rice varieties. With this method, we quantitively analyzed genotypic differences in grain-filling ability and used the grain weight distribution parameters for genomic prediction subject to genetic improvement in grain yield in rice. The novel description method could represent the observed grain weight distribution with five genotype-specific parameters of a mixture of two gamma distributions. The estimated genotype-specific parameters representing the proportion of filled grains had applicability to explain the grain filling ability of genotypes comparable to that of sink-filling rate and the conventionally measured proportion of filled grains, which suggested the efficiency and flexibility of grain weight distribution parameters to handle several genotypes. We revealed that perfectly filled grains have to be prioritized over partially filled grains for the optimum allocation of the source of yield in a panicle, from the analysis for obtaining an ideal shape of grain weight distribution. We conducted genomic prediction of grain weight distribution considering five genotype-specific parameters of the distribution as phenotypes relating to grain filling ability. The proportion of filled grains, average weight of filled grains, and variance of filled grain weight, which were considered to control grain yield to a certain degree, were predicted with accuracies of 0.30, 0.28, and 0.53, respectively. The proposed description method of grain weight distribution facilitated not only the investigation of the optimum allocation of nutrients in a panicle for realizing high grain-filling ability, but also allowed genomic selection of grain weight distribution.

## Introduction

The demand for an increase in food production has risen dramatically because of world population explosion and climate change [1,2]. Genomics-based breeding may facilitate genetic improvements leading to higher crop yields [3]. Genomic selection (GS) [4] is one of the most promising methods to improve polygenic traits, such as crop yield [5]. In GS, a prediction model is built based on the information from a training population with phenotypic data (dependent variable) and genome-wide marker data (independent variables), and genotypic values of genotyped breeding materials (i.e. test population) are predicted using the prediction model [6]. Both simulation studies [7,8] and real breeding programs [9,10] demonstrated that GS could increase genetic gain in crop breeding. In a conventional breeding strategy, the information on yield-related traits is considered to be more applicable for improving crop yields [11]. The information on yield-related traits (i.e. secondary traits) could also improve accuracy and efficiency of GS for predicting grain yield (i.e. target trait) [10,12]. The efficient accumulation of the information on the secondary traits into the GS strategy will dramatically increase the genetic gain of complex target traits.

Rice (*Oryza sativa* L.) is a staple grain especially in Asia [13]. The characteristics of grain filling (e.g., grain-filling rate, variation of grain weights in a plant, and process of single-grain growth) partially determine the grain yield of rice. Recently developed high-yielding rice varieties have been selected to show high sink capacity (total number of grains × filled grain weight), but their grain-filling ability varies considerably [14,15]. The genetic improvement in grain-filling characteristics and their proper integration into a selection model will increase genetic gain in grain yield. When the available source of yield is limited for a rice plant, it is important to efficiently partition them into grains in a panicle. The variations in the grain-filling speed and nutrient partitioning among grains cause dispersion in grain weights, and they are genotype- and environment-dependent [16,17,18,19]. Thus, in this study, we developed a novel method to use grain-filling characteristics (i.e. the degree of dispersion in grain weights) as a secondary trait when the main target trait was grain yield of rice in GS.

Grain weight distribution can simultaneously represent the degree of dispersion in grain weights and the percentage of filled grains. Grain weight distribution generally shows a bimodal distribution composed of the peak of unfilled/partially filled grains and the peak of filled grains [19]. In the present study, we developed a method to describe the grain weight distribution to quantitatively represent this trait. The previously proposed methods (Gini coefficient and coefficient of variation [20]) are not suitable for describing the entire shape of a grain weight distribution because they do not consider the bimodal distribution. Although Miyagawa (1980) introduced a method to describe grain weight distribution in soybean (*Glycine max* (L.) Merr.), considering the mixture of exponential distribution and Gaussian distribution [21], this method was not suitable when the number of samples (i.e. genotypes) was large because of the need for counting 0-mg values (i.e. degenerated organ or sterile flowers). Herein, we introduce a novel description method to assess grain weight distribution to carry out GS for grain weight distribution. We treated the genotype-specific parameters estimated for describing grain weight distribution as the target traits representing the characteristics of grain filling.

The main purposes of this study were i) to develop a method to describe grain weight distribution and ii) to evaluate the efficiency of genomic prediction for the genotype-specific parameters of grain weight distribution. First, to evaluate the efficiency of the description method for assessing grain weight distribution, we analyzed genotypic differences in grain-filling ability based on the genotype-specific parameters of grain weight distribution. Second, we evaluated the accuracy of genomic prediction for the genotype-specific parameters of the grain weight distribution. Because grain weight distribution is one of the traits that can be measured in the final growth stage, it is necessary to predict the grain weight distribution parameters (i.e. secondary traits) instead of using observed values when we assume the prediction of grain yield (i.e. target trait) at an early stage of growth. Based on these analyses, we evaluated the efficiency of grain weight distribution as the secondary trait of grain yield.

## Materials and methods

### Field experiment

In the present study, 128 Japanese rice cultivars (S1 Table) were cultivated in the Yawara experimental field at the National Agriculture and Food Research Organization at Tsukubamirai, Ibaraki, Japan (36°00′N, 140°02′E), in 2015. These cultivars’ seeds were collected as Kobe collection in Kobe University. Twenty-two-day-old seedlings were transplanted on 14 May, one plant per hill, with a density of 22.2 (15 × 30 cm) hills per square meter. Two blocked replications were prepared, in each of which two sub-blocks were made. Each cultivar was planted in a row once in a replication, and the cultivars were arranged by consecutive number in a sub-block. The position of sub-blocks within a replication and the order of cultivars within a sub-block were reversed between replications, so that the effect of the direction on each genotype differed between two replications. A fertilization regime of N:P_2_O_5_:K_2_O = 5:5:5 g/m^2^ was applied as a basal dressing. Sixty percent of N was applied as a controlled release fertilizer. The cultivation was conducted using established management practices for field preparation and the control of pest and diseases.

The heading date, on which 50% of panicles emerged in a population, was recorded for each cultivar. After the maturity, four panicles were sampled for each cultivar (i.e. two panicles were sampled in a replication). The number of panicle samples of Nangokusodachi (Variety 10) was three because of the lack of unbroken panicles in the paddies. In the sampling practice, one superior panicle was sampled from a plant. The total grain number per panicle was measured and recorded. Unhulled grains were dried at 80°C for 72 hours to enable the measurement of grain weight on an absolutely dry matter basis. The total grain weight for each genotype was measured on a dry matter basis, after which single grain weights were measured using an automatic counting and weighing system (QWCALC, NK-Systems, Aichi, Japan), and the resultant single grain weights were adjusted using the total grain weight to obtain the dry matter weights. We assumed the differences in grain weight distributions were related only to cultivar differences, because the difference in the grain weight distributions between two replications in each cultivar was little. Thus, the following analyses were conducted after we pooled the data from two replications for each cultivar. As the position of a grain on a panicle is known to be related to the single grain weight [22,23], it is informative to link the single grain weight to its position on a panicle. We measured the single grain weights of two grains attached to specific positions on a panicle (i.e. superior and inferior grains). They were sampled from the middle primary branch in a panicle. A superior grain was sampled from the bottom from among the grains attaching directly to the primary branch, while an inferior grain was the second grain from the top on the bottommost secondary branch on the primary branch.

Additionally, to evaluate the relationship between the novel proposed parameters, which we describe later, and the conventional traits related to grain-filling characteristics, we also measured the proportion of unfilled grains in other two different ways (*p*.*23mg* and *p*.*90%*) and the sink-filling rate. *p*.*23mg* was the probability of grains being lighter than 23 mg, which was determined based on conventional evaluation for cultivars for grain weight. Although the threshold of filled-grain weight might depend on the cultivars in conventional evaluation, we decided to use the threshold of 23 mg in order to set a single threshold for filled-grain weight. *p*.*90%* was the probability of grains being lighter than 90% of the weight at the 95^th^ percentile of the grain weight distribution. The sink-filling rate was calculated as the ratio of total grain weight to sink capacity. For the calculation of sink capacity, the value at the 95^th^ percentile of the observed grain weight distribution was multiplied by the number of grains for each cultivar.

### Marker genotype

Marker genotype data was available for 123 cultivars (S1 Table). DNA was extracted from one typical individual plant from each cultivar using the CTAB method [24]. Next-generation sequencing data was obtained using the Illumina HiSeq 2000, HiSeq 4000 and HiseqX systems via paired-end sequencing (Illumina, Inc., San Diego, CA, USA). Data sets deposited in the DDBJ Sequence Read Archive (DRA002219, SRA106223, DRA000158, DRA000307, DRA000897, DRA000927, DRA004358, ERA358140) were reanalyzed. Adapters and low-quality bases were removed from paired reads using the Trimmomatic v0.36 program [25]. The preprocessed reads were aligned on the Os-Nipponbare-Reference-IRGSP-1.0 [26] by using the bwa-0.7.12 mem algorithm with the default options [27]. SNP calling was based on alignment using the Genome Analysis Toolkit (GATK, 3.7-0-gcfedb67 [28]) and Picard package V2.5.0 (http://broadinstitute.github.io/picard). The mapped reads were realigned using RealignerTargetCreator and indelRealigner of GATK software. SNPs and InDel were called at the population level using the UnifiedGenotyper of GATK with the -glm BOTH option. We used only biallelic non-missing sites over all cultivars with a minor allele frequency (MAF) ≥ 0.025.

For further analysis, genotypes were represented as -1 (Homozygous Reference), 1 (Homozygous Alternate) or 0 (Heterozygous). Markers with a proportion of heterozygous loci less than 5% were used because the phenotyped and genotyped plants were not identical, owing to the assumption that almost all markers were well fixed in rice cultivars after several generations of self-pollination. We selected 42,508 markers that were polymorphic and showed incomplete linkage with other makers.

### Description of grain weight distribution

We assumed that the difference in grain weight distribution among cultivars could be represented by the difference in the distribution parameters. We approximated grain weight distribution using a mixture of gamma distributions. The probability density of a single seed weight *x* in a cultivar was represented as:

$$f\left(x\right)=px^{a_{1}-1}\frac{exp\left(-\frac{x}{b_{1}}\right)}{\Gamma \left(a_{1}\right)b_{1}{}^{a_{1}}}+\left(1-p\right)x^{a_{2}-1}\frac{exp\left(-\frac{x}{b_{2}}\right)}{\Gamma \left(a_{2}\right)b_{2}{}^{a_{2}}}$$ [1]

where *p* was the mixing proportion, *a*_1_ and *a*_2_ were shape parameters, *b*_1_ and *b*_2_ were scale parameters of the two gamma density functions, and Γ(.) represented a gamma function. The first (left-side) gamma distribution represents the distribution of unfilled grains and the second (right-side) gamma distribution the distribution of filled grains.

The distribution parameters, *a*_1_, *a*_*2*_, *b*_1_, and *b*_2_, were not easy to understand intuitively. Therefore, we used feature values of the distribution (mode of left-side distribution: *mode1 =* (*a*_1_*−*1)*b*_1_, skewness of left-side distribution: *skewness1 =* 2 */* (*b*_1_^1/2^), mean of right-side distribution: *mu2 = a*_2_*b*_2_, and variance of right-side distribution: *var2 = a*_2_*b*_2_^2^) to interpret the shape of the grain weight distributions. The parameter *p* could be interpreted as the percentage of unfilled grains (i.e., the value of (1 –*p*) is the percentage of filled grains).

### Parameter estimation for grain weight distribution

We estimated the distribution parameters using the expectation-maximization (EM) algorithm [29]. For the estimation of gamma distribution parameters, we used the method developed by Minka (2000; 2002) [30,31]. The initial value of *p* was set to 0.5. For the initial values of *a*_1_, *a*_2_, *b*_1_, and *b*_2_,

$$a_{0}=\frac{{x^{\prime }}^{2}}{Variance\left(x\right)}$$ [2]

$$b_{0}=\frac{Variance\left(x\right)}{x^{\prime }}$$ [3]

were used. We sampled two single grains and used their weight as *x*′; the lighter one was used for the initial values of *a*_1_ and *b*_1_, and the heavier one was used for the initial values of *a*_2_ and *b*_2_. *Variance(x)* was the variance of the weights of grains in a cultivar. We used the EM algorithm 100 times with different initial values of *a*_1_, *a*_2_, *b*_1_, and *b*_2_ and different samples for *x*′ in each cultivar. The single parameter set with the highest log-likelihood was defined as comprising cultivar-specific parameters. The analysis was conducted using R [32].

### Genomic prediction

To build a model that predicted grain weight distribution parameters (i.e., *p*, *a*_*1*_, *a*_*2*_, *b*_*1*_, and *b*_*2*_) based on the genome-wide markers mentioned above, we applied two methods: genomic best linear unbiased prediction (GBLUP) and partial least squares (PLS) regression. For GBLUP, we built a model for each distribution parameters, generating five prediction models for one prediction trial. To build the model, we used the function ‘kinship.BLUP’ in the ‘rrBLUP’ package [33] in R. For PLS regression, the model could predict multiple parameters simultaneously [34]. We assumed three types of PLS regression models. For the first type (1 parameter-group PLS), we built a single PLS regression model to predict the set of grain weight distribution parameters, so that all five grain weight distribution parameters were predicted simultaneously. For the second type (3 parameter-group PLS), we built three PLS regression models–the first for the parameter *p*, the second for the parameters *a*_*1*_ and *b*_*1*_, representing the left-side distribution, and the third for the parameters *a*_*2*_ and *b*_*2*_, representing the right-side distribution, because it was expected that the left-side and right-side distributions and their mixing coefficient could be controlled in different manners. For the third type (each-parameter PLS), we built five PLS regression models to predict the five distribution parameters separately. To determine the number of PLS components, we conducted leave-one-out cross validation. The PLS components with the highest accuracy (i.e., Q^2^ explained below) were used. The maximum number of PLS components was set to 30. To build the model, we used the function ‘plsr’ in the ‘pls’ package [35].

To evaluate the potential effectiveness of the model for genomic prediction, model accuracy was assessed. Prediction ability was evaluated using leave-one-out cross validation. For the distribution parameters, accuracy was calculated using Pearson’s correlation coefficient between observed and fitted/predicted values.

In addition to the accuracy of the distribution parameters, we evaluated the accuracy of the “shapes” of distributions. The predicted residual error sum of square (PRESS) for the mixture of gamma distributions (i.e., the grain weight distribution) was calculated by summing the squared prediction error of grain weight distribution over the cultivars, thus:

$$\begin{array}{l} PRESS=\sum_{l}\left\{\int_{0}^{\infty }{\left[f_{l}\left(x\right)-\hat{f_{l}\left(x\right)}\right]}^{2}dx\right\}\\ \;=\sum_{l}\{p_{l}^{2}g\left(a_{l1},b_{l1}\right)+{\left(1-p_{l}\right)}^{2}g\left(a_{l2},b_{l2}\right)+{\hat{p}}_{l}^{2}g\left({\hat{a}}_{l1},{\hat{b}}_{l1}\right)\\ \;+{\left(1-{\hat{p}}_{l}\right)}^{2}g\left({\hat{a}}_{l2},{\hat{b}}_{l2}\right)+p_{l}\left(1-p_{l}\right)h\left(a_{l1},a_{l2},b_{l1},b_{l2}\right)\\ \;-p_{l}{\hat{p}}_{l}h\left(a_{l1},{\hat{a}}_{l1},b_{l1},{\hat{b}}_{l1}\right)-p_{l}\left(1-{\hat{p}}_{l}\right)h\left(a_{l1},{\hat{a}}_{l2},b_{l1},{\hat{b}}_{l2}\right)\\ \;-\left(1-p_{l}\right){\hat{p}}_{l}h\left(a_{l2},{\hat{a}}_{l1},b_{l2},{\hat{b}}_{l1}\right)-\left(1-p_{l}\right)\left(1-{\hat{p}}_{l}\right)h\left(a_{l2},{\hat{a}}_{l2},b_{l2},{\hat{b}}_{l2}\right)\\ \;-{\hat{p}}_{l}\left(1-{\hat{p}}_{l}\right)h\left({\hat{a}}_{l1},{\hat{a}}_{l2},{\hat{b}}_{l1},{\hat{b}}_{l2}\right)\} \end{array}$$ [4]

where

$$g\left(\alpha ,\beta \right)=\frac{2^{1-2\alpha }}{\left(2\alpha -1\right)\beta }\frac{1}{B\left(\alpha ,\alpha \right)}$$ [5]

$$h\left(\alpha_{1},\alpha_{2},\beta_{1},\beta_{2}\right)=\frac{2\beta_{1}^{\alpha_{2}-1}\beta_{2}^{\alpha_{1}-1}}{\left(\alpha_{1}+\alpha_{2}-1\right){\left(\beta_{1}+\beta_{2}\right)}^{\alpha_{1}+\alpha_{2}-1}}\frac{1}{B\left(\alpha_{1},\alpha_{2}\right)}$$ [6]

In theabove equation, *f*_*l*_(*x*) represents the observed probability density function in the cultivar *l* (*l* = 1, …, 99), $\hat{f_{l}(x)}$ is the probability density function predicted by a prediction model, and *B*(., .) is the beta function. *p*_*l*_, *a*_*l*1_, *a*_*l*2_, *b*_*l*1_, and *b*_*l*2_ are observed distribution parameters in the cultivar *l*, and ${\hat{a}}_{l1},\;{\hat{a}}_{l2},\;{\hat{b}}_{l1}$, and ${\hat{b}}_{l2}$ are the predicted parameters. The accuracy of the predicted shape of grain weight distribution for a cultivar population was measured as:

$$Q^{2}=1-\frac{PRESS}{\sum_{l}\left\{\int_{0}^{\infty }{\left[f_{l}\left(x\right)-\bar{f_{l}\left(x\right)}\right]}^{2}dx\right\}}$$ [7]

where $\sum_{l}\left\{\int_{0}^{\infty }{\left[f_{l}(x)-\bar{f_{l}(x)}\right]}^{2}\;dx\right)$ could be calculated by substituting ${\hat{a}}_{l1},\;{\hat{a}}_{l2},\;{\hat{b}}_{l1}$, and ${\hat{b}}_{l2}$ in Eq 4 with ${\bar{a}}_{l1},\;{\bar{a}}_{l2},\;{\bar{b}}_{l1}$, and ${\bar{b}}_{l2}$ (i.e., the observed means of parameters over all cultivars), respectively.

## Results

### Description of grain weight distribution

**Fig 1:**
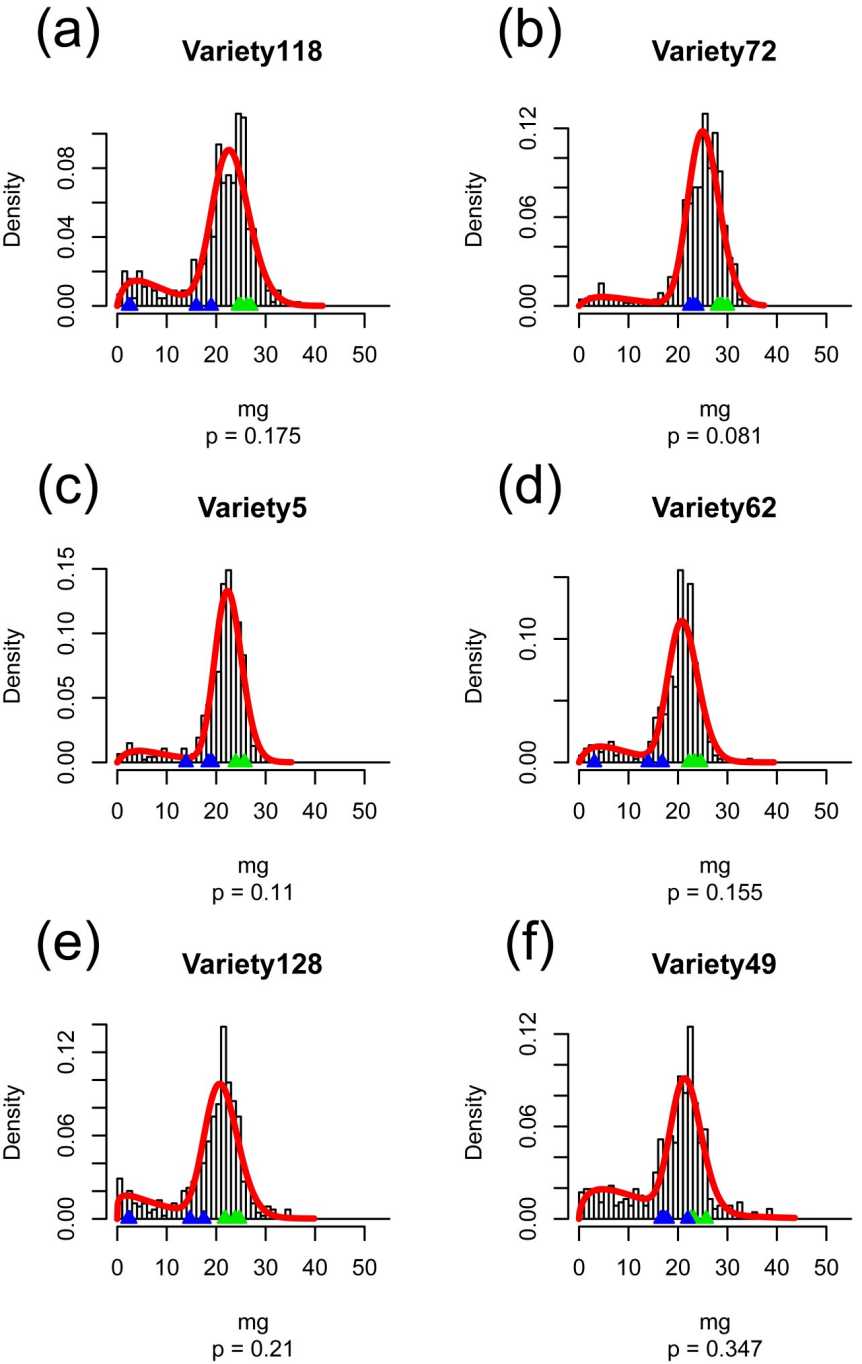
Histogram and estimated probability density function (red line) of grain weight in six cultivars. Blue and green points represent the weight of inferior and superior grain derived from the center primary branch on each panicle. The parameter ‘*p*’ indicates (a) mean (0.175), (b) < 5% (0.081), (c) 25% (0.110), (d) 50% (0.155), (e) 75% (0.210), and (f) 95% (0.347) for all cultivars.

**Fig 2:**
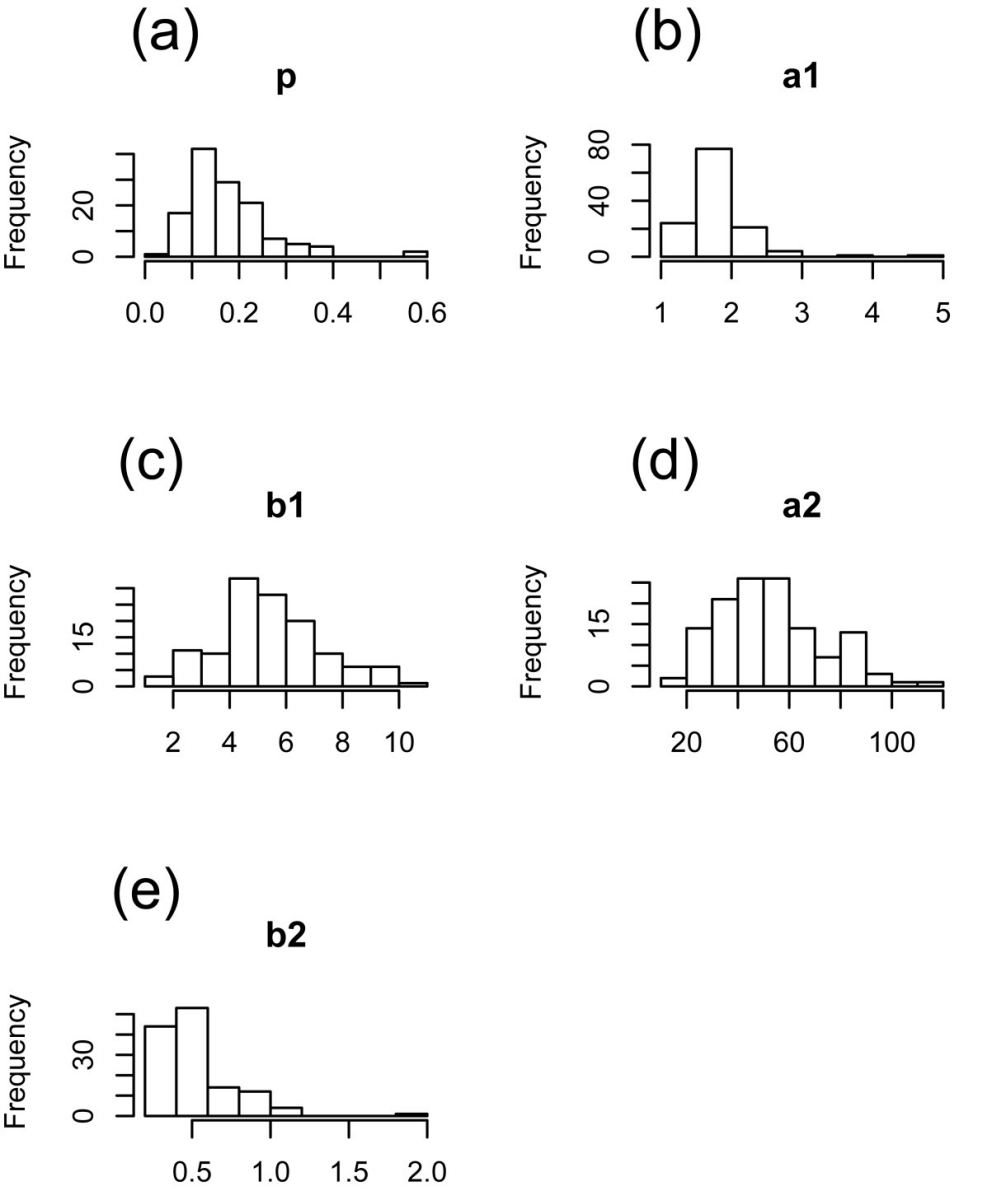
Histogram of estimated grain weight distribution parameters. (a) *p*, (b) *a*_1_, (c) *b*_1_, (d) *a*_2_, and (e) *b*_2_ in Eq 1 are shown.

The description for grain weight distribution (Eq 1) could estimate the variation of grain weight in each cultivar (Fig 1 and S1 Fig). When the parameter estimation trials with different initial values were conducted 100 times, the same estimates were obtained, suggesting that our estimation found the global solution for each cultivar. A jagged histogram of observed grain weight might be due to the small data size for each cultivar. The estimated distributions matched with the observed ones. The estimated distribution parameters showed variation among 128 cultivars (Fig 2). There were two cultivars (Variety 37 and Variety97) for which more than half of the grains were classified into the unfilled grain distribution (i.e. the estimated *p* was larger than 0.5 in these cultivars) (Fig 2A). The grains of Variety 97 were not clearly separated into two gamma distributions (S1 Fig).

**Fig 3:**
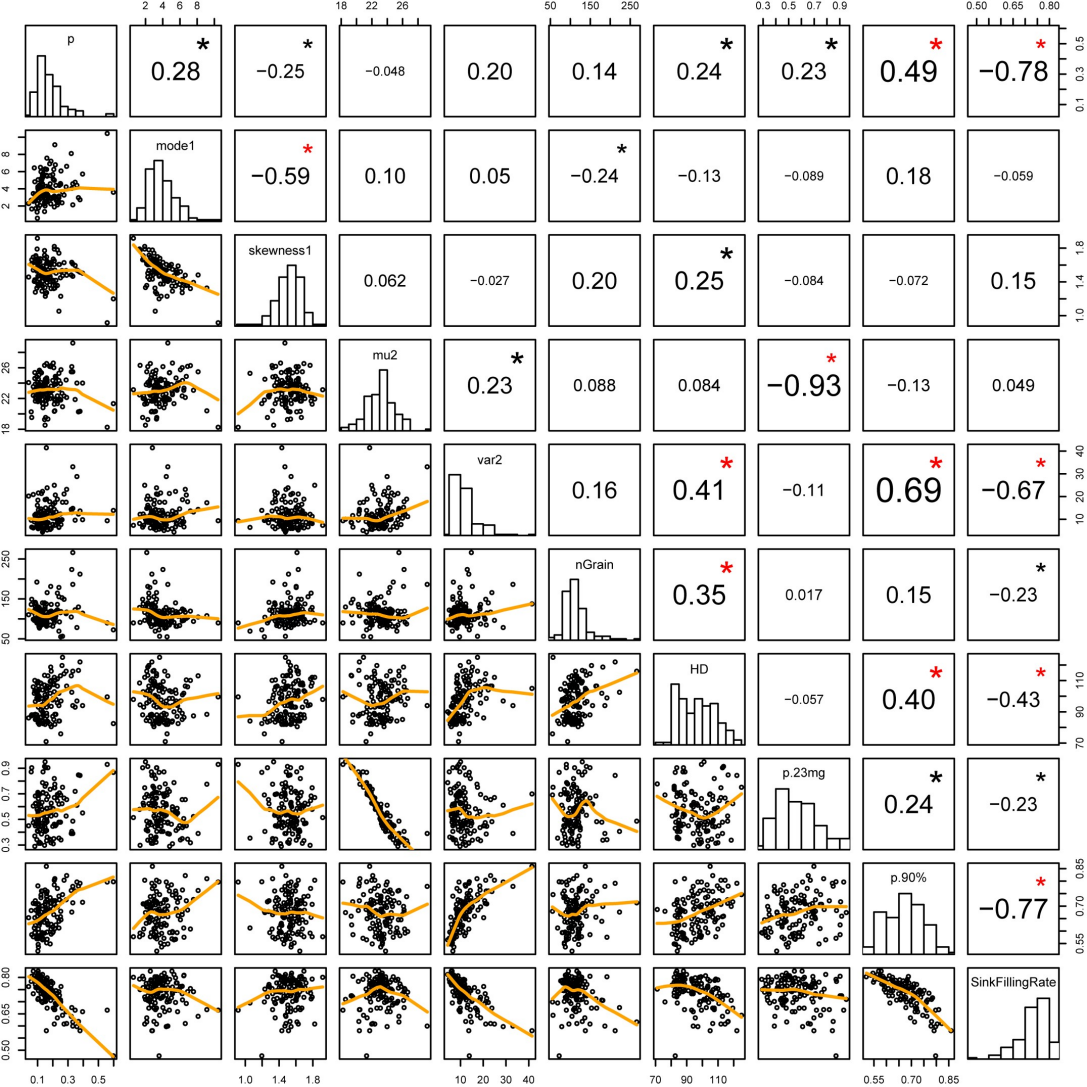
Relations among feature values of grain weight distribution and panicle traits in examined cultivars. The diagonal panel shows histograms of each trait. Lower and upper triangle panels show the scatter plot and correlation coefficient between the two traits. Black and red asterisks show the significance at 1% and 0.1%, respectively, in correlation test. nGrain: average number of grains per panicle, HD: days from sowing to heading, p.23mg: probability of grains that were lighter than 23mg, p.90%: probability of grains that were lighter than 90% weight of the weight at 95% point of the grain weight distribution, SinkFillingRate: sink-filling rate using the weight at 95% point of the grain weight distribution in each cultivar as the sink capacity of one grain.

Days to heading positively correlated with *var2* and the number of grains per panicle (Fig 3; p-value < 0.01 in the correlation test). A significant negative correlation (p < 0.001) was observed between *mode1* and *skewness1*, in which the combination of large *mode1* and small *skewness1* showed a symmetric left-side gamma distribution (i.e. unfilled grain distribution), and the combination of small *mode1* and large *skewness1* showed the right-skewed left-side gamma distribution. For the traits used in conventional breeding, the sink-filling rate showed a significant negative correlation with *p*, *var2*, number of grains per panicle, days to heading, *p*.*23mg*, and *p*.*90%*. The *p*.*90%*, which showed a significant negative correlation with sink-filling rate, showed a significant positive correlation with traits that correlated with sink-filling rate, except for the number of grains per panicle. A negative correlation between the sink-filling rate and the days to heading was observed especially in cultivars that required more than 100 days from germination to heading (the fourth column from the right on the bottom row in Fig 3). The *p*.*23mg* showed a significant negative correlation with *mu2*. Although significant positive correlations (p < 0.001) between days to heading and the number of grains and *var2* were observed, the correlation between the number of grains and *var2* was not significant.

**Fig 4:**
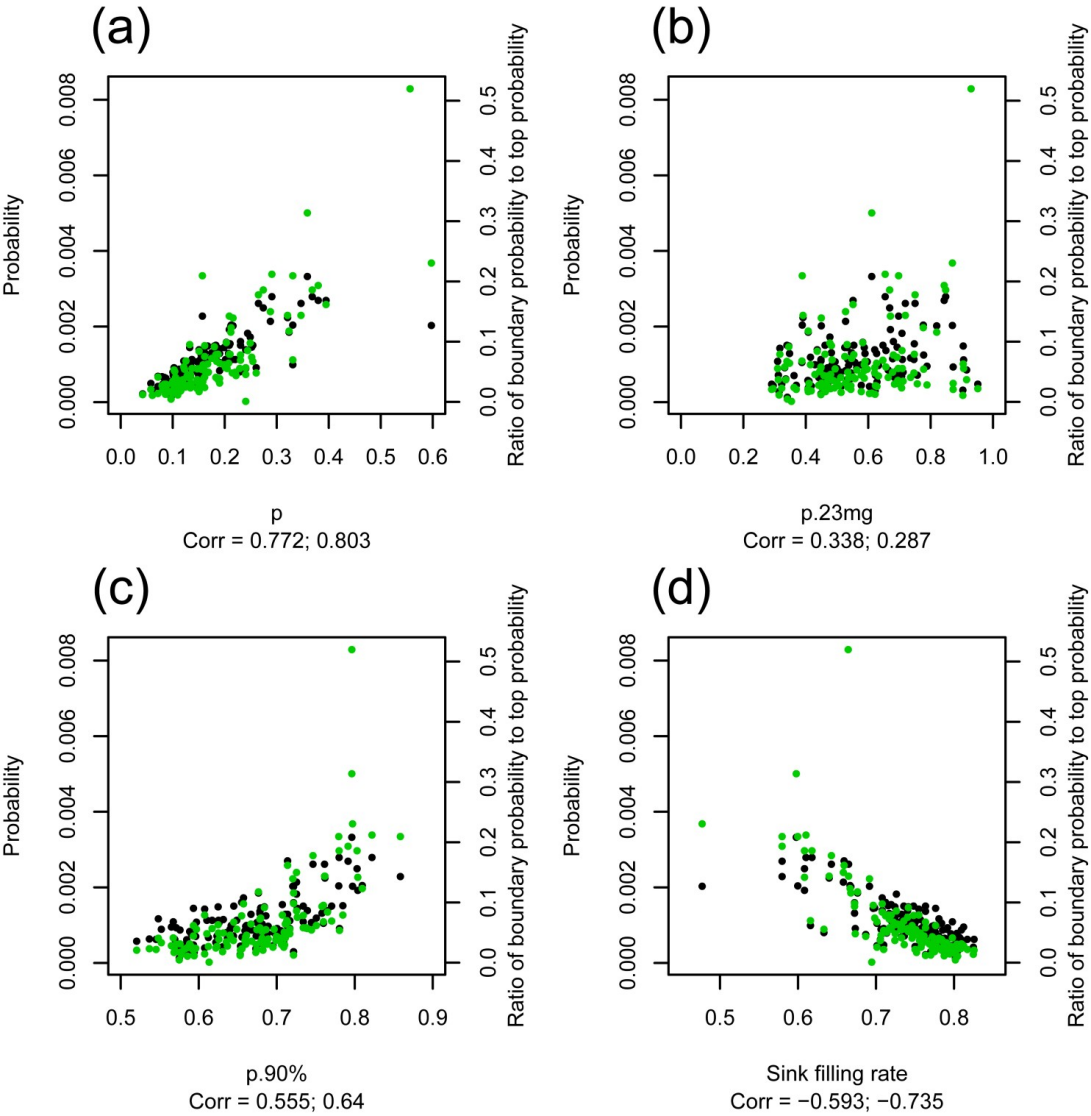
Relation between the distinguishability of left-side and right-side gamma distributions of grain weight distribution and the distribution parameter *p* (a), the probability of grains that were lighter than 23 mg (b), the probability of grains that were lighter than 90% weight of the weight at the 95% point of the grain weight distribution (c), and the sink-filling rate using the weight at the 95% point of the grain weight distribution for each cultivar considering the sink capacity of one grain (d). Black points represent the probability around the boundary point ±0.1 mg in the grain weight distribution. Green points represent the ratio of the probability around the boundary point ±0.1 mg to the probability around the mode of the right-side gamma distribution ±0.1 mg.

To evaluate the distinguishability of the left-side and right-side gamma distributions in the grain weight distribution, we calculated the probability around the boundary of the two gamma distributions from the estimated grain weight distribution. The point with the lowest density between the peaks of the left-side and right-side gamma distributions was defined as the boundary point. Grain weight at the boundary point positively correlated with the parameter *mu2* (*r* = 0.68) (S2 Fig). The probability around the boundary point (± 0.1mg; boundary probability) positively correlated with the parameter *p* (*r* = 0.77), *p*.*23mg* (*r* = 0.34), and *p*.*90%* (*r* = 0.56) (Fig 4A–4C). The boundary probability negatively correlated with the sink-filling rate (*r* = -0.60) (Fig 4D). The ratio of the boundary probability to the probability around the mode of the right-side gamma distribution, which represents the distinguishability of the left-side and right-side gamma distributions more intuitively than the boundary probability, showed a more significant correlation with *p* (*r* = 0.80), *p*.*90%* (*r* = 0.64), *p* (*r* = -0.74). The results showed that the cultivars with more filled grains had a more clearly distinguishable boundary between filled and unfilled grain distributions. The grouping of cultivars based on the distribution parameters (i.e. *p*, *a*_1_, *b*_1_, *a*_2_, and *b*_2_) corresponded to the sink-filling rate (Part A in S3 Fig). The group showing a high sink-filling rate also had a sharp filled grain distribution and a clearly distinguishable boundary between filled and unfilled grain distributions (Part B in S3 Fig).

In most cultivars, superior grains were more heavy than inferior grains (S1 Fig). Among all examined cultivars, 91.7% of superior grains and 72.3% of inferior grains sampled from middle primary branches were classified into right-side gamma distribution (i.e. filled grains). The correlations between the grain weight distribution parameter *p* and the average number of grains per panicle classified into unfilled grains were 0.41 and 0.35 for superior and inferior grains, respectively.

### Genomic prediction

In the 1 parameter-group PLS, the number of PLS components required to represent the largest Q^2^ among all tested number of components in the leave-one-out cross validation was determined to be 3. In the 3 parameter-group PLS, the number of PLS components was 1, 3, and 3 for the model of *p*, *a*_1_ and *b*_1_, *a*_2_ and *b*_2_, respectively. In the each-parameter PLS, the number of PLS components was 3, 4, 3, 1, and 1 for the model of *p*, *a*_1_, *b*_1_, *a*_2_, and *b*_2_, respectively.

**Fig 5:**
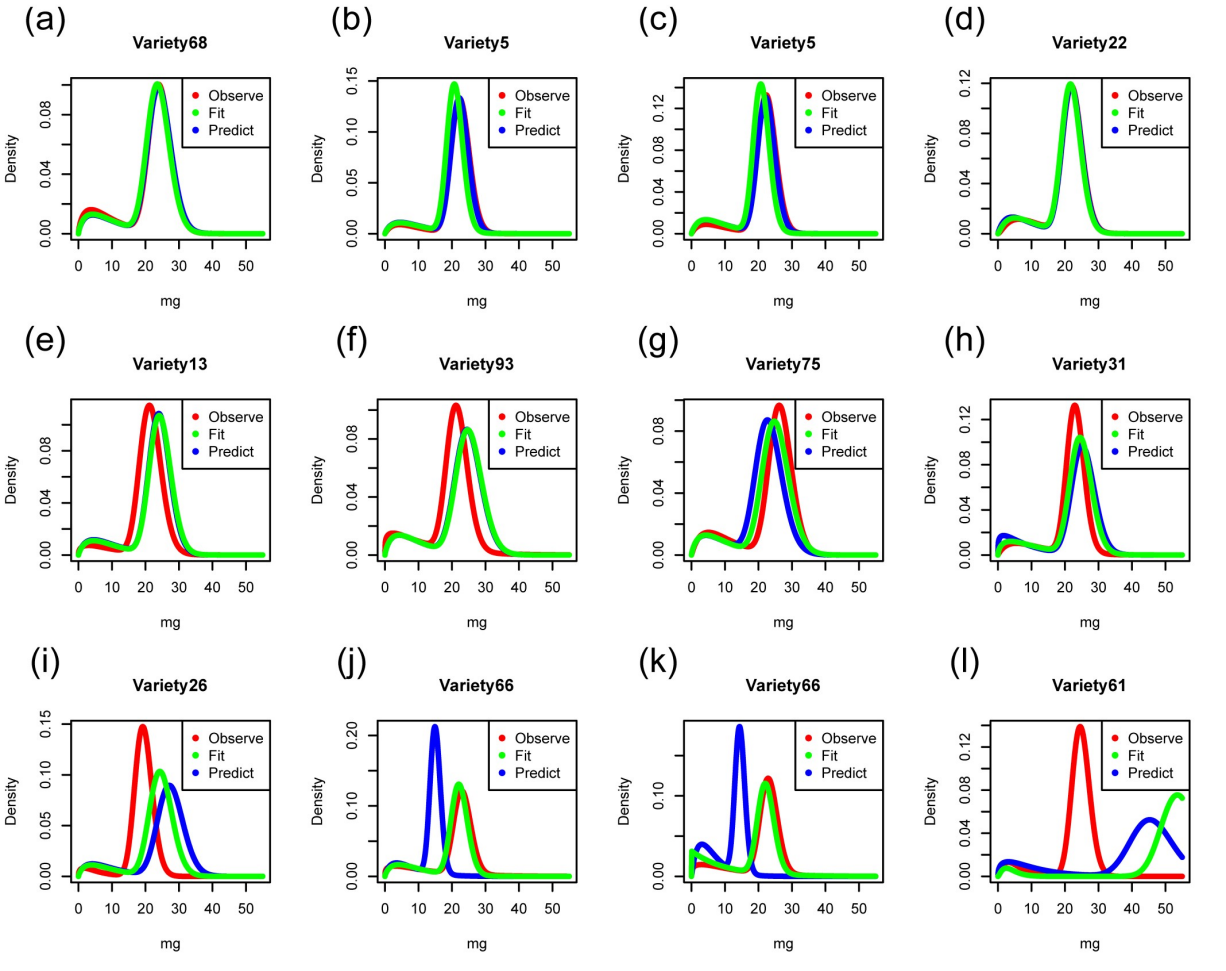
Examples of the observed probability density function (red line) of the grain weight compared with fitted (green) and predicted (blue) ones that result from genomic prediction for weight distribution parameters. The predicted squared residual error of each distribution was (a-d) minimum, (e-h) mean, and (i-l) maximum in GBLUP (a, e, and i), PLS using 1 parameter-group (b, f, and j), PLS using 3 parameter-group (c, g, and k), and PLS using each-parameter group (d, h, and l).

The shape of grain weight distribution was predicted using four methods (Fig 5 and S4 Fig). The prediction accuracy in all cultivars (Q^2^) was higher in PLS than in GBLUP, and did not differ considerably among the three PLS regression models (Table 1). The direction of the gap between observed and predicted distribution was dependent on prediction method even for one variety in some cases, e.g., Variety1 and Variety40 (S4 Fig). In all prediction methods, the prediction accuracy in *a*_1_ and *b*_1_ was quite low, while the prediction accuracy in *a*_2_ and *b*_2_ was more than 0.3, except for that in *a*_2_ by each-parameter PLS (Table 2). For *p*, the each-parameter PLS attained the highest prediction accuracy among all methods, but the accuracy was just 0.3. In GBLUP, predicted values shrank to the mean value for all distribution parameters. Although the fitted accuracy of all distribution parameters was greater than 0.5 in GBLUP, their prediction accuracy decreased drastically, especially for the parameters representing left-side distribution (i.e. *a*_1_ and *b*_1_). In the 1 parameter-group PLS and 3 parameter-group PLS, the predicted values shrank to the mean value for *p*, *a*_1_, and *b*_1_. These two PLS methods predicted a similar shape of the right-side distribution (i.e. the values of *a*_2_ and *b*_2_) (S4 Fig); thus, the accuracies of these parameters in the 1 parameter-group PLS and the 3 parameter-group PLS were same (Table 2). The each-parameter PLS showed the least shrinkage to the mean value for all distribution parameters. The prediction accuracy of the feature values of grain weight distribution (i.e. *mode1*, *skewness1*, *mu2*, and *var2*) showed that all prediction methods could well predict the variance of the right-side distribution (i.e. *var2*), except for the each-parameter PLS, but they could not predict the shape of the left-side distribution (i.e. *mode1* and *skewness1*). By GBLUP, the prediction accuracy of the feature values did not change largely when the predicted values were calculated using predicted distribution parameters (i.e. *p*, *a*_*1*_, *b*_*1*_, *a*_*2*_, and *b*_*2*_) from when the prediction model was built for each feature value. We found that all methods could not predict new distribution-related traits (i.e. boundary point and boundary probability between unfilled and filled grain distribution). The prediction model built to predict the rank of boundary probability among varieties was not successful (*r* = -0.36 in GBLUP). The ratio of boundary probability to the mode of the right-side distribution and its rank could not be predicted accurately (*r* = 0.05 and 0.15, respectively). Genomic prediction was not suitable for the prediction of distinguishability between unfilled and filled grain distribution. For the conventionally measured traits (i.e. *p*.*23mg*, *p*.*90%*, and sink-filling rate), prediction of *p*.*23mg* was possible by GBLUP and each-parameter PLS, but the accuracy was still low, and prediction of *p*.*90%* was possible in GBLUP, 1 parameter-group PLS, and 3 parameter-group PLS (from 0.264 to 0.271). The prediction accuracy of sink-filling rate that was conducted directly by GBLUP (*r* = 0.406) was higher than that of other conventional traits.

**Table 1:** PRESS and prediction accuracy (Q^2^) for the grain weight distribution in genomic prediction.

	Fitting				Prediction			
	GBLUP	PLS (1 group)	PLS (3 groups)	PLS (each)	GBLUP	PLS (1 group)	PLS (3 groups)	PLS (each)
PRESS	2.448	2.616	2.594	2.259	3.007	2.697	2.666	2.553
Q2	0.366	0.323	0.329	0.415	0.222	0.302	0.310	0.339

**Table 2:** Accuracy (correlation coefficient between observed and fitted/predicted values) for the parameters of grain weight distribution in genomic prediction. The predicted values of feature values of distribution and the conventional traits was calculated using the predicted distribution parameters. The accuracy in parentheses is that from the prediction conducted for the trait itself.

	Fitting accuracy				Prediction accuracy			
	GBLUP	PLS (1 group)	PLS (3 groups)	PLS (each)	GBLUP	PLS (1 group)	PLS (3 groups)	PLS (each)
p	0.727	0.330	0.326	0.777	0.217	0.181	0.174	0.300
a1	0.555	0.139	0.463	0.821	-0.055	0.004	0.141	0.133
b1	0.637	0.052	0.788	0.789	-0.266	-0.307	0.130	0.128
a2	0.703	0.727	0.727	0.483	0.337	0.371	0.371	0.243
b2	0.842	0.732	0.732	0.646	0.463	0.422	0.422	0.358
mode1	0.435 (0.372)	0.085	0.132	0.624	-0.146 (-0.520)	0.004	0.050	-0.019
skewness1	0.534 (0.597)	0.132	0.498	0.769	-0.074 (-0.041)	-0.011	0.161	0.140
mu2	0.337 (0.773)	0.110	0.110	0.309	0.280 (0.257)	-0.002	-0.002	0.215
var2	0.876 (0.888)	0.668	0.668	0.573	0.533 (0.530)	0.466	0.466	0.260
boundary point	0.042 (0.577)	0.155	0.247	0.014	0.006 (0.137)	0.092	0.099	-0.156
boundary probability	0.318 (0.479)	0.249	0.273	0.220	-0.116 (-0.766)	-0.004	-0.006	0.163
p.23mg	0.222 (0.682)	-0.057	-0.043	0.296	0.163 (0.154)	-0.051	-0.045	0.144
p.90%	0.579 (0.641)	0.565	0.582	0.087	0.264 (0.193)	0.268	0.271	-0.079
sink-filling rate	(0.812)	-	-	-	(0.406)	-	-	-

**Fig 6:**
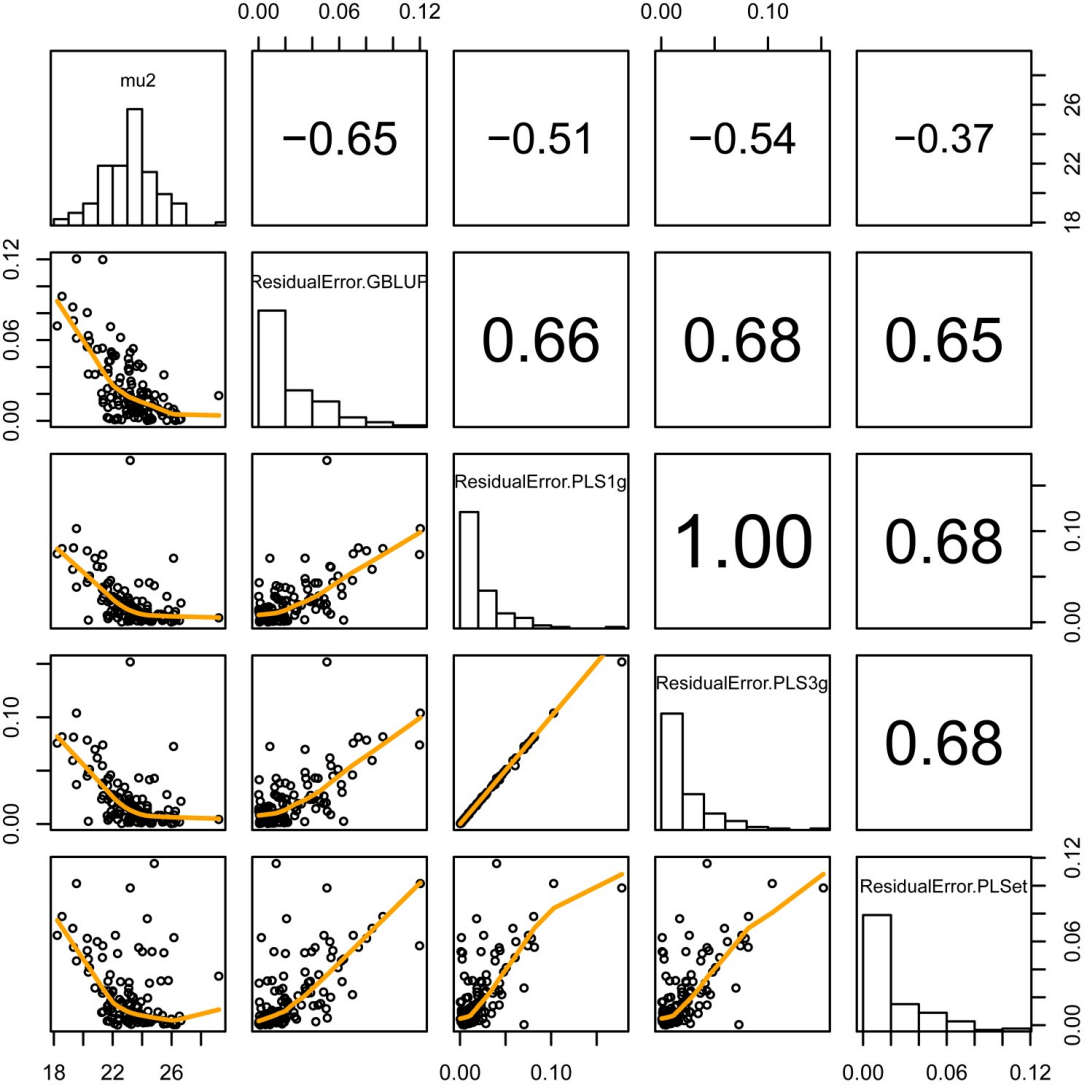
Relation between predicted squared residual error in each cultivar and observed parameter of grain weight distribution, *mu2*. The diagonal panel shows histograms of each variable. The lower and upper triangle panels show the scatter plot and correlation coefficient between two variables. ResidualError.GBLUP, ResidualError.PLS1g, ResidualError.PLS3g, and ResidualError.PLSet represent the predicted squared residual errors in GBLUP, PLS using 1 parameter-group, PLS using 3 parameter-groups, and PLS using each-parameter group, respectively.

The predicted squared residual error in each cultivar varied among the examined cultivars (top left of Fig 6). The prediction success for genotypes was not dependent on genomic prediction method (4 × 4 boxes of right bottom in Fig 6). Cultivars with small *mu2* (i.e. mean weight in filled grains) showed a tendency to show large prediction error.

## Discussion

The method of the description of grain weight distribution could depict the observed grain weight distribution in a rice population (Fig 1 and S1 Fig). Although the sudden convex and concave patterns could not be caught by the description method (e.g., Fig 1A and 1E), our description method is more reliable and balanced than conventional histograms because the sudden ruggedness in histograms owing to small sample sizes would disturb the subsequent analyses. In our description methods, we assumed that the rice grain weight distribution is bimodal. However, the bimodality of the distribution for Variety 48 and Variety 97 was not clear (S1 Fig). It is possible that some genotypes might result in unimodal or flat grain weight distribution in both favorable and unfavorable environments [18]. Although our description method was suitable for our current data, it is necessary to deal with both unimodal and bimodal distributions in multi-environmental experiments (especially when extreme environments are included). We can solve this problem by additionally visually or statistically (likelihood) comparing the fitness of the estimated probability density function to the measured histogram between bimodal and unimodal distribution.

Each estimated parameter of grain weight distribution varied among genotypes (Fig 2) and represented various shapes of grain weight distribution (Fig 1 and S1 Fig). We supposed that these parameters could be treated as quantitative traits. The use of feature values of the distribution (i.e. *mode1*, *skewness1*, *mu2*, and *var2*) also helped us to understand the shape of the distribution. To evaluate the efficiency of the genotype-specific parameters relating to grain weight distribution in rice breeding, we checked the relationship between the distribution parameters and traits that are used in conventional breeding. *p*.*23mg*, which had been considered to be compatible with the distribution parameter *p*, showed only moderate correlation with the distribution parameter *p* because *p*.*23mg* depended on the grain size (i.e. *mu2*), while *p*.*90%* and sink-filling rate showed high correlation with *p* (Fig 3). The boundary point of left-side and right-side distribution also showed a significant positive correlation with *mu2* (S2 Fig). These results suggest that it is difficult to define an identical threshold of filled grain weight for a large number of cultivars, and that the genotype-specific parameter *p* could serve as a flexible indicator of grain-filling ability when handling a large number of genotypes.

In almost all cultivars, there were some unfilled grains on a panicle (Fig 2A). Although we could not determine why more than half of the grains were assigned to the left-side distribution in the two cultivars (Variety 37 and 97), it is possible that a giant-embryo mutation, which was introduced by chance in Variety 97 in this season, resulted in the high percentage of unfilled grains. Fig 4 suggests that the cultivars with poor grain-filling ability produced more half-filled grains, i.e. the distinguishability of the boundary between the left-side and right-side distributions was poor (S3 Fig). The cultivars that can produce more ‘completely filled’ grains without producing partially filled grains are preferred even if they produce some percentage of almost-empty husks, when the source of yield is limited. We may assume that the cultivars with poor grain-filling ability might also be poor at partitioning their limited source among grains. The method for describing grain weight distribution revealed that the optimal grain weight distribution shape with limited source was one in which the right-side distribution was sharp and the boundary probability was low.

Homogeneity in grain weight is one of the important traits, i.e., a small value of *var2*, is preferred. In our data, the average weight of filled grains (i.e. *mu2*) did not showed significant correlation with the proportion of filled grains nor the sink-filling rate, suggesting that the average of filled grain weight were not affected by the ability of allocation of the source in a panicle in a single environment. The cultivars that required more days from germination to heading had a tendency to show larger variation among the weights of grains in the right-side distribution (i.e. *var2*) and lower sink-filling rate, especially for cultivars whose heading dates were after late August (Fig 3). One of the reasons might be the lower temperature in September and October (21.6°C and 17.0°C on average, respectively) (The Agro- Meteorological Grid Square Data [36]). We thought that a delayed heading date (long vegetative stage) would increase the value of parameter *var2* owing to the production of a large number of grains in a panicle. However, the Pearson’s correlation between number of grains and *var2* was not significant (Fig 3), and the partial correlation between number of grains and *var2* given heading date was quite low (*ρ* = 0.02), suggesting that the number of grains did not affect the dispersion of grain weight, so that the ability of allocation of the source in a panicle did not depend on the number of grains. In the present study, we also showed that the grains that bloomed later (i.e. inferior grains) were lighter than those that bloomed earlier (i.e. superior grains) even in one primary branch in a panicle (S1 Fig). It is known that the variation in flowering date among spikelets in a panicle results in variation in the grain weight in a panicle for a cultivar [22,16], and that the speed of blooming among grains in a panicle is genotype-dependent [37]. It is possible that single grain weight varied largely in cultivars whose blooming timing varied, but the relationship between the weight of inferior/superior grains and the speed of blooming should be evaluated in more detail to verify their causal relationship.

In the present study, we conducted a genomic prediction for grain weight distribution treating the genotype-specific parameters of grain weight distribution as phenotypes. The distribution related genotype-specific parameters, *p*, *a*_2_, and *b*_2_ (hence, the feature parameters *mu2* and *var2*) could be predicted by the GBLUP and each-parameter PLS methods (Table 2). In rice breeding, the important traits that related to grain weight distribution are the proportion of filled grains and the characteristics of filled grains, which are represented by *p*, *mu2*, and *var2*. Our results showed the possibility of genomic prediction for traits representing grain-filling ability.

GBLUP showed the highest prediction accuracy of *mu2* and *var2* among the four methods and the second highest accuracy in predicting *p* (Table 2). However, in the previous studies, the effectiveness of the multiple-trait genomic prediction model especially for highly correlated traits has been demonstrated [38,39]. Our results contrast those previously reported despite the high correlation between our target traits (correlation coefficient between *p* and *a*_1_ was 0.34, between *a*_1_ and *b*_1_ was -0.53, and between *a*_2_ and *b*_2_ was -0.83; Fig 3). The previous study demonstrated the efficiency of multiple-trait GBLUP, which explicitly involved the correlation between traits in the model structure, and showed that GBLUP worked well for predicting traits with low heritability when the other traits had high heritability [38]. We used PLS regression for multiple-trait genomic prediction, which did not involve phenotypic correlation explicitly in the model structure but considered PLS score to explain all traits. Thus, results similar to multiple-trait GBLUP were expected for PLS regression. However, in our study, because each group of distribution parameters with high phenotypic correlation consisted of parameters with similar prediction accuracy, the multiple-trait prediction model did not improve prediction accuracy.

Each-parameter PLS showed highest prediction accuracy of the total shape of grain weight distribution, whereas all PLS methods showed similar accuracy (Table 1). The result suggests that the PLS method was useful for predicting the shape of grain weight distribution using multiple parameters. Here, the 1 parameter-group PLS and 3 parameter-group PLS predicted the same value for the parameters describing the right-side gamma distribution (i.e. *a*_2_ and *b*_2_) (Fig 5 and S4 Fig). In PLS regression, the scores were estimated to obtain high correlation between scores for response and explanatory variables [40]. The estimated scores and loadings of PLS were approximately equal using these two PLS models (correlation coefficient > 0.99), possibly because 1 parameter-group PLS attached too much importance to the prediction for *a*_2_ and *b*_2_. The prediction method that shows the highest accuracy would depend on the situation. For example, Xu et al. (2018) reported that PLS showed the worst predictability in six prediction methods they examined, which included GBLUP, for rice agronomic traits [41], while Burstin et al. (2015) reported that PLS could performed better than other methods in some traits [42]. Although Iwata and Jannink (2011) also showed that PLS attained lower prediction accuracy than other prediction methods (i.e. ridge regression, Bayesian methods, and other methods) in their simulation studies, they could find that a meta-predictor that combined PLS regression with other methods could attained higher accuracy than the best single method in their examined methods [43]. Their results suggested that PLS could capture additional information of relationship between markers and phenotypes that the other methods could not capture. PLS provides the linear relationship between phenotypic values and marker genotypes, but it explains predictor variables by small number of latent variables unlike GBLUP. Thus, PLS might capture the pleiotropic effects for instance, which worked linearly but could not be captured by GBLUP well, while the further studies will be needed to clarify what aspects of information could be captured by PLS. In the present study, moreover, the prediction accuracy of shape, Q^2^, was used for deciding the number of PLS components in the present PLS regression model. If we decided the number of PLS components based on the prediction accuracy of each distribution parameter instead of Q^2^ only, the chosen number of PLS components were different. For example, the number of PLS components that showed the highest accuracy for predicting *p* was five in each-parameter PLS (*r* = 0.347), while we chose three PLS components based on Q^2^. The PLS methods may be preferred to predict the shape of grain weight distribution when we decide the number of PLS components based on Q^2^. The purpose of the present genomic prediction was to predict the genotype-specific parameters that related to grain yield; thus *p*, *mu2*, and *var2* were the main targets. However, the entire shape of grain weight distribution will be highly important when we model the partitioning of the source of yield in a panicle. In such cases, a high prediction accuracy of the entire shape of the distribution may be helpful.

We have planned to use grain weight distribution as the secondary trait to model grain yield (main trait). The prediction accuracy of the main trait in GS depends on the reliability of secondary trait prediction [38,12]. This is also suggested by the selection index theory [44]. Therefore, improvement in the prediction accuracy of secondary traits (grain weight distribution parameters) may be desired. The predictability of the grain weight distribution varied among cultivars (S1 Fig). Prediction for the cultivars with small grains was difficult (Fig 6) because of the small sample size of genotypes with small grain in the training population to accurately predict genotypes having small grains. Another possible reason might be the low prediction ability for outlier when the predicted values largely shrink to the mean value in the training population, especially when the accuracy is not so high [10]. It is known that the difference in the population structure between test and training populations also has an impact on prediction accuracy [45]. However, we could not find any relationship between accuracy and population structure in our data (S5 Fig). Our leave-one-out cross validation results suggest that the population structure did not result in a bias in predictability among cultivars in the present study. The low prediction ability, especially in left-side distribution parameters, might be because unpollinated spikelets were not separated from unfilled/partially filled grains and because the distribution of the weights of partially filled grains largely depends on environmental factors [46,18], which might cause quite low heritability in the left-side distribution parameters in our data. In fact, the narrow-sense heritabilities based on the genetic and error variances estimated in GBLUP were 0.146, 0.004, 0.054, 0.21, and 0.409 for *p*, *mode1*, *skewness1*, *mu2*, and *var2*, respectively. This result shows the low heritability in left-side distribution relating traits, and thus the difficulty in the prediction of traits with low heritability. For example, temperature [47], amount of solar radiation [46], and nitrogen supply [48] affect the grain yield. The panicle structure also affects the grain weight distribution [49]. Thus, these data will improve the prediction ability of grain weight distribution. Jarquin et al. (2014), Malosetti et al. (2016) and Bustos et al. (2016) have proposed different types of methods to include environmental covariates in the genomic prediction model [50,51,52]. In the next step, information on environmental factors and growth process of rice plant would be needed to improve the predictability in grain yield.

## Conclusion

The method proposed in the present study could successfully describe grain weight distribution in rice using five genotype-specific parameters. Based on the genotype-specific parameters, we could analyze the genotypic differences in grain-filling-related traits as quantitative traits in Japanese rice cultivars. The description method enabled us to estimate the optimal shape of grain weight distribution that could be realized in our examined population, considering the allocation of the source of yield in a panicle. These genotype-specific parameters of grain weight distribution could contribute as target traits in GS. The important traits for rice breeding, the proportion of filled grains, average weight of filled grains and dispersion in the weight of filled grains could be predicted by using GBLUP. The information on grain weight distribution-related traits can be expected to improve grain yield in rice.

## Supporting information

S1 Table Cultivars examined in the present study. Of the 128 cultivars, 123 cultivars had marker genotype data based on their resequencing data, which were shown in the column of ‘Marker genotype’ as “O”.
(PDF)

S1 Fig Histogram and estimated probability density function (red line) of grain weight. Blue and green points represent the weight of inferior and superior grain derived from the center primary branch on each panicle.
(PDF)

S2 Fig Relation between the grain weight at the boundary point in the grain weight distribution and the distribution parameter *mu2*. (PDF)

S3 Fig **Principal component analysis of grain weight distribution parameters among 128 cultivars (a) and representative grain weight distribution of each cultivar group (b).** Cultivars were grouped by the k-means method using grain weight distribution parameters. Each color represents each cultivar group, and the same color is used for a specific cultivar group both in (a) and (b). Point size in (a) represents the sink-filling rate in each cultivar. In (b), the red solid line shows the distribution based on the median of each parameter among all cultivars. The dashed lines show the distribution using the median of each parameter in each cultivar group.
(PDF)

S4 Fig **Probability density function (left) and cumulative probability (right) of observed and predicted grain weight distribution.** Left-side plot among two plots with the same Variety ID shows the result of comparison between the observed probability density function (red line) of grain weight and predicted ones (blue). Right-side plot shows the result of comparison between cumulative probability of observed and predicted grain weight distribution. These results were derived from genomic prediction for grain weight distribution parameters by GBLUP (solid line), PLS using 1 parameter-group (dashed line), and PLS using 3 parameter-group (dotted line).
(PDF)

S5 Fig Principal component analysis using genome-wide markers among 128 cultivars. (a) The X and Y-axes show the scores of PC1 and PC2, respectively. (b) The X and Y-axes show the scores of PC1 and PC3, respectively. The size of the points represents the predicted residual error in the prediction by GBLUP for each cultivar.
(PDF)

## References

[1] Tester M, Langridge P. Breeding technologies to increase cropping production in a changing world. Science. 2010; 327: 818–822. 10.1126/science.1183700 20150489 10.1126/science.1183700

[2] Alexandratos N, Bruinsma J. World agriculture towards 2030/2050: the 2012 revision. FAO: ESA Working Paper No. 12–03. 2012.

[3] Abberton M, Batley J, Bentley A, Bryant J, Cai H, Cockram J, et al Global agricultural intensification during climate change: a role for genomics. Plant Biol. J. 2016; 14: 1095–1098. 10.1111/pbi.12467 PMC5049667 26360509

[4] Meuwissen THE, Hayes BJ, Goddard ME. Prediction of total genetic value using genome-wide dense marker maps. Genetics. 2001; 157: 1819–1829. 11290733 10.1093/genetics/157.4.1819 PMC1461589

[5] Bernardo R. Bandwagons I, too, have known. Theor. Appl. Genet. 2016; 129:2323–2332. 10.1007/s00122-016-2772-5 27681088 10.1007/s00122-016-2772-5

[6] Heffner EL, Sorrells ME, Jannink J-L. Genomic selection for crop improvement. Crop Sci. 2009; 49: 1–12.

[7] Jannink J-L. Dynamics of long-term genomic selection. Genet. Sel. Evol. 2010; 42: 35 10.1186/1297-9686-42-35 20712894 10.1186/1297-9686-42-35 PMC2936280

[8] Mayor PJ, Bernardo R. Genomewide selection and marker-assisted recurrent selection in doubled haploid versus F2 populations. Crop Sci. 2009; 49: 1719–1725.

[9] Beyene Y, Semagn K, Mugo S, Tarekegne A, Babu R, Meisel B, et al Genetic gains in grain yield through genomic selection in eight bi-parental maize populations under drought stress. Crop Sci. 2015; 55: 154–163.

[10] Yabe S, Hara T, Ueno M, Enoki H, Kimura T, Nishimura S, et al Potential of genomic selection in mass selection breeding of an allogamous crop: an empirical study to increase yield of common buckwheat. Frontiers Plant Sci. 2018; 9: 276 10.3389/fpls.2018.00276 29619035 10.3389/fpls.2018.00276 PMC5871932

[11] Reynolds MP, Quilligan E, Aggarwal PK, Bansal KC, Cavalieri AJ, Chapman SC, et al An integrated approach to maintaining cereal productivity under climate change. Global Food Security. 2016; 8: 9–18.

[12] Rutkoski J, Poland J, Mondal S, Autrique E, Perez LG, Crossa J, et al Canopy temperature and vegetation indices from high-throughput phenotyping inprove accuracy of pedigree and genomic selection for grain yield in wheat. G3. 2016; 6: 2799–2808. 10.1534/g3.116.032888 27402362 10.1534/g3.116.032888 PMC5015937

[13] FAOSTAT. 2013; http://www.fao.org/faostat/en/#data/FBS. Accessed 22 August 2017.

[14] Yang J, Zhang J. Grain-filling problem in ‘super’ rice. J. Exp. Bot. 2009; 61: 1–5. 10.1093/jxb/erp348 19959608

[15] Yoshinaga S, Takai T, Arai-Sanoh Y, Ishimaru T, Kondo M. Varietal differences in sink production and grain-filling ability in recently developed high-yielding rice (*Oryza sativa* L.) varieties in Japan. Field Crops Res. 2013; 150: 74–82.

[16] Yang J, Zhang J, Wang Z, Liu K, Wang P. Post-anthesis development of inferior and superior spikelets in rice in relation to abscisic acid and ethylene. J. Exp. Bot. 2006; 57: 149–160. 10.1093/jxb/erj018 16330527 10.1093/jxb/erj018

[17] Jiang Q, Du Y, Tian X, Wang Q, Xiong R, Xu G, et al Effect of panicle nitrogen on grain filling characteristics of high-yielding rice cultivars. Europ. J. Agronomy. 2016; 74: 185–192.

[18] Tsukaguchi T, Murakami K, Michimoto T. A quantitative measure for assimilate partitioning efficiency in rice (*Oryza sativa* L.). Field Crops Res. 2016; 198: 122–130.

[19] Okamura M, Arai-Sanoh Y, Yoshida H, Mukouyama T, Adachi S, Yabe S, et al Characterization of high-yielding rice cultivars with different grain-filling properties to clarify limiting factors for improving grain yield. Field Crops Res. 2018; 219: 139–147.

[20] Du Y-J, Li Z-Z, Li W-L. Effect of different water supply regimes on growth and size hierarchy in spring wheat populations under mulched with clear plastic film. Agric. Water Manage. 2006; 79(3): 265–279.

[21] Miyagawa S. Variation on the frequency distribution of the single-seed-weight of soybean. Japan. J. Breed. 1980; 30(3): 260–271. (written in Japanese with English summary.)

[22] Hoshikawa K. The growing rice plant: an anatomical monograph Tokyo: Nobunkyo 1989. (written in Japanese)

[23] Kuwano M, Takaiwa F, Yoshida KT. Differential effects of a transgene to confer low phytic acid in caryopses located at different positions in rice panicles. Plant Cell Physiol. 2009; 50(7): 1387–1392. 10.1093/pcp/pcp071 19465440 10.1093/pcp/pcp071

[24] Murray MG, Thompson WF. Rapid isolation of high molecular weight plant DNA. Nucleic Acid Research. 1980;8: 4321–4326. 10.1093/nar/8.19.4321 PMC324241 7433111

[25] Bolger A M, Lohse M, Usadel B. Trimmomatic: A flexible trimmer for Illumina Sequence Data. Bioinformatics 2014; btu170. 10.1093/bioinformatics/btu170 PMC4103590 24695404

[26] Kawahara Y, De la Bastide M, Hamilton JP, Kanamori H, McCombie WR, Ouyang S, et al Improvement of the *Oryza sativa* Nipponbare reference genome using next generation sequence and optical map data. Rice. 2013; 6: 4 10.1186/1939-8433-6-4 24280374 10.1186/1939-8433-6-4 PMC5395016

[27] Li H. Exploring single-sample SNP and INDEL calling with whole-genome de novo assembly. Bioinformatics 2012; 28: 1838–1844. 10.1093/bioinformatics/bts280 22569178 10.1093/bioinformatics/bts280 PMC3389770

[28] McKenna A, Hanna M, Banks E, Sivachenko A, Cibulskis K, Kernytsky A, et al The Genome Analysis Toolkit: a MapReduce framework for analyzing next-generation DNA sequencing data. Genome Res. 2010;20: 1297–303. 10.1101/gr.107524.110 20644199 10.1101/gr.107524.110 PMC2928508

[29] Dempster AP, Laird NM, Rubin DB. Maximum likelihood from incomplete data via the EM algorithm. J Royal Stat. Soc, B. 1977; 39(1): 1–38.

[30] Minka TP. Estimating a Dirichlet distribution. 2000; https://tminka.github.io/papers/dirichlet/minka-dirichlet.pdf.

[31] Minka TP. Estimating a Gamma distribution Microsoft Research, Cambridge, UK, Tech. Rep. 2002; https://tminka.github.io/papers/minka-gamma.pdf.

[32] R Core Team. R: A language and environment for statistical computing R Foundation for Statistical Computing, Vienna, Austria https://www.R-project.org/.

[33] Endelman JB. Ridge regression and other kernels for genomic selection with R package rrBLUP. Plant Genome. 2011; 4(3): 250–255.

[34] Iwata H, Ebana K, Uga Y, Hayashi T. Genomic prediction of biological shape: elliptic frontier analysis and kernel partial least squares (PLS) regression applied to grain shape prediction in rice (*Oryza sativa* L.). PLoS ONE. 2015; 10(3): e0120610 10.1371/journal.pone.0120610 25825876 10.1371/journal.pone.0120610 PMC4380318

[35] Mevik BH, Wehrens R. The pls package: principal component and partial least squares regression in R. J. Stat. Softw. 2007; 18(2): 1–23.

[36] NIAES. The Agro- Meteorological Grid Square Data. http://mesh.dc.affrc.go.jp/opendap/. Accessed 22 August 2017.

[37] Yabe S, Nakagawa H, Adachi S, Mukouyama T, Arai-Sanoh Y, Okamura M, et al Model analysis of genotypic difference in the variation of the duration from heading to flower opening based on the flower position on a panicle in high-yielding rice cultivars. Field Crops Res. 2018; 223: 155–163.

[38] Jia Y, Jannink J-L. Multiple-trait genomic selection methods increase genetic value prediction accuracy. Genetics. 2012; 192: 1513–1522. 10.1534/genetics.112.144246 23086217 10.1534/genetics.112.144246 PMC3512156

[39] Wang X, Li L, Yang Z, Zheng X, Yu S, Xu C, et al Predicting rice hybrid performance using univariate and multivariate GBLUP models based on North Carolina mating design II. Heredity. 2017; 118: 302–310. 10.1038/hdy.2016.87 27649618 10.1038/hdy.2016.87 PMC5315526

[40] Hastie T, Tibshirani R, Friedman J. The element of statistical learning New York: Springer 2001.

[41] Xu Y, Wang X, Ding X, Zheng X, Yang Z, Xu C, et al Genomic selection of agronomic traits in hybrid rice using an NCII population. Rice. 2018; 11: 32 10.1186/s12284-018-0223-4 29748895 10.1186/s12284-018-0223-4 PMC5945574

[42] Burstin J, Salloignon P, Chabert-Martinello M, Magnin-Robert J-B, Siol M, Jacquin F, et al Genetic diversity and trait genomic prediction in a pea diversity panel. BMC Genomics. 2015; 16:105 10.1186/s12864-015-1266-1 25765216 10.1186/s12864-015-1266-1 PMC4355348

[43] Iwata H, Jannink J-L. Accuracy of genomic selection prediction in barley breeding programs: a simulation study based on the real single nucleotide polymorphism data of barley breeding lines. Crop Science. 2011; 4:1915–27.

[44] Falconer DS, Mackay TFC. Introduction to quantitative genetics 4th edn Pearson/Prentice Hall; 1996.

[45] Minamikawa MF, Nonaka K, Kaminuma E, Kajiya-Kanegae H, Onogi A, Goto S, et al Genome-wide association study and genomic prediction in citrus: potential of genomics-assisted breeding for fruit quality traits. Sci. Rep. 2017; 7: 4721 10.1038/s41598-017-05100-x 28680114 10.1038/s41598-017-05100-x PMC5498537

[46] Nagato K, Chaudhry FM. Influence of panicle clipping, flag leaf cutting and shading on ripening of japonica and indica rice. Japanese J. Crop Sci. 1970; 39(2): 204–212.

[47] Kotaka S, Abe N. Varietal differences of air temperature during ripening period of rice. J. Agr. Met. 1981; 37 (3): 245–248. (written in Japanese)

[48] Yoshida H, Horie T. A model for simulating plant N accumulation, growth and yield of diverse rice genotypes grown under different soil and climatic conditions. Field Crops Res. 2010; 117: 122–130.

[49] Kato T. Panicle centroid index: an index to represent the distribution of spikelets in a rice panicle. Kinki. J. Crop Sci. Breed. 2006; 51: 31–36.

[50] Jarquin D, Crossa J, Lacaze X, Cheyron PD, Daucourt J, Lorgeou J, et al A reaction norm model for genomic selection using high-dimensional genomic and environmental data. Theor. Appl. Genet. 2014; 127: 595–607. 10.1007/s00122-013-2243-1 24337101 10.1007/s00122-013-2243-1 PMC3931944

[51] Malosetti M, Bustos-Korts D, Boer MP, van Eeuwijk FA. Predicting responses in multiple environments: issues in relation to genotype x environment interactions. Crop Sci. 2016; 56: 2210–2222.

[52] Bustos-Korts D, Malosetti M, Chapman S, van Eeuwijk F. Modelling of genotype by environment interaction and prediction of complex traits across multiple environments as a synthesis of crop growth modelling, genetics and statistics In: Yin X, Struik PC, editors. Crop systems biology. Springer; 2016 pp. 55–82. 10.1186/s12918-016-0289-9

