# Supplementary material for: Description of grain weight distribution leading to genomic selection for grain-filling characteristics in rice
Source: PLoS One. 2018 Nov 20;13(11):e0207627. doi: 10.1371/journal.pone.0207627 (PMC6245794; doi:10.1371/journal.pone.0207627)
Supplement: S1 Table — Of the 128 cultivars, 123 cultivars had marker genotype data based on their resequencing data, which were shown in the column of ‘Marker genotype’ as “O”. (PDF) [file pone.0207627.s001.pdf]

Supplementary Table S1 Cultivars examined in the present study. Of the 128 cultivars, 123 cultivars had marker genotype data based on their resequencing data, which were shown in the column of 'Marker genotype' as "O".

No.	Variety name	Improved line / landrace	Pedigree	Marker genotype
1	Tsugaruroman	Improved	Fukei 141/Akitakomachi	O
2	Yumeakari	Improved	Akitakomachi/Aokei 110	O
3	Mutsuhomare	Improved	Todoroki-wase/Akihikari//Fuji 329	O
4	Fukei175	Improved	Hitomebore/Fukei 141	O
5	Akihikari	Improved	Toyonishiki/Reimei	O
6	Reimei	Improved	Mutant from Fujiminori irradiated by gamma-ray	O
7	Fujisaka5	Improved	Futaba/Zengokuwase	O
8	Natsuhikari	Improved	Katsurawase/Seinan 45	O
9	Kamenoo4	*Landrace*	Selection form Kamenoo	O
10	Nangokusodachi	Improved	Kouiku30/Koukei265	O
11	Toyonishiki	Improved	Sasanishiki/Ou 239	O
12	Ouu197	Improved	Ban 33/Kamenoo 4	O
13	Chiyohonami	Improved	Akihomare/Koganehikari	O
14	Hitomebore	Improved	Koshihikari/Hatsuboshi	O
15	Manamusume	Improved	Chiyonishiki/Hitomebore	O
16	Akitakomachi	Improved	Koshihikari/Ou 292	O
17	Okiniiri	Improved	Chubu 47/Ou 313	O
18	Fukuhibiki	Improved	Kochihibiki/Ou 316	O
19	Himenomochi	Improved	Daikei 227/Koganemochi	O
20	Otomemochi	Improved	Koganemochi/Yamasenishiki	O
21	Benisengoku	Improved	Tokaiasahi/Norin 26	O
22	Haenuki	Improved	Shonai 29/Akitakomachi	O
23	Domannaka	Improved	Ibukiwase/Shonai 29	O
24	Rikuu132	Improved	Rikuu 20/Kamenoo 4	O
25	Joushu	*Landrace*		O
26	Senichi	*Landrace*		O
27	Hatsunishiki	Improved	Norin 22/Norin 1	O
28	Yamasenishiki	Improved	Norin 22/Norin 1	O
29	Sasashigure	Improved	Norin 8/Tohoku 24	O
30	Sasanishiki	Improved	Hatsunishiki/Sasashigure	O
31	Tsuyahime	Improved	Yamagata70/Touhoku164	O
32	Moeminori	Improved	Nankai128/Haenuki	O
33	Kinuhikari	Improved	Shu 2800/Hokuriku 100//Nagoyutaka	O
34	Dontokoi	Improved	Kinuhikari/Hokuriku 120	O
35	Itadaki	Improved	Dontokoi/Shu 4695	O
36	Nourin1	Improved	Morita-wase/Rikuu 132	O
37	Hounen-wase	Improved	Norin 22/Norin 1	O
38	Todoroki-wase	Improved	Shu 921/Honen-wase	O
39	Koshihikari	Improved	Norin 22/Norin 1	O
40	Hanaechizen	Improved	Etsunan 122/Fukuhikari	O
41	Yukinosei	Improved	Fukou 101/Niigata 8	O
42	Gohyakumanngoku	Improved	Kikusui/Shin 200	O
43	Koganemochi	Improved	Sinanomochi 3/Norin 17	O
44	Koshiji-wase	Improved	Norin 22/Norin 1	O
45	Fusaotome	Improved	Hitomebore/Hanaechizen	O
46	Mineasahi	Improved	Kanto 79/Kihou	O
47	Yumetsukushi	Improved	Kinuhikari/Koshihikari	O
48	Kihou	Improved	GinfujiB/Akibare	O
49	Akanezora	Improved	Tsukinohikari/Koshihikari	O
50	Hatsuboshi	Improved	Koshihikari/Kihou	O
51	Chiyonishiki	Improved	Hatsuboshi/Toyonishiki	O
52	Menkoina	Improved	Hitomebore/Akita 39	O
53	Notohikari	Improved	Fukuhikari/Koshiji-wase	O
54	Hohohonoho	Improved	Notohikari/Akitakomachi	O
55	Haruru	Improved	Yamahoushi/Koshihikari	O
56	Rikuu20	*Landrace*	Pure line selection from Aikoku	O
57	Sekitori	*Landrace*		O
58	Mizuhonokagayaki	Improved	Hokuriku174/Chubu98	O
59	Eminokizuna	Improved	Gikei120/Shu6602	O
60	Akisakari	Improved	Awaminori/Etsunan173	O

61	Hokuriku193	Improved	Jo344/Keicho2	O
62	Kantou IL1	Improved	Koshihikari	O
63	Kantou IL3	Improved	Koshihikari	O
64	Goropikari	Improved	Tsukinohikari/Koshihikari	O
65	Nihonmasari	Improved	Kochikaze/Nipponbare	O
66	Takanari	Improved	Mitsuyou42/Mitsuyou25	O
67	Mirenishiki	Improved	Hinohikari/Inakei 517	O
68	Satojiman	Improved	Kanto 175/Etsunan 154	O
69	Aikoku	*Landrace*		O
70	Ohba	*Landrace*		O
71	Kamenoo	*Landrace*		O
72	Futaba	Improved	Shinju 2/Takaneasahi	O
73	Nipponbare	Improved	Yamabiko/Sachikaze	O
74	Koganebare	Improved	Nipponbare/Kihou	O
75	Taichung65	Improved	Kameji/Shinriki	O
76	Yumehitachi	Improved	Chiyonishiki/Kinuhikari	
77	Asahinoyume	Improved	Aichinokaori//Tskinohikari/Aichi 65	O
78	Nourin6	Improved	Joshu/Senichi	O
79	Nourin8	Improved	Ginbozu/Asahi	O
80	Momiroman	Improved	IR65598-112-2/Seikai203	O
81	Akidawara	Improved	Mirenishiki/Ikuhikari	O
82	Yamadawara	Improved	Izumi348/Tokai192	O
83	Hatsushimo	Improved	Higashiyama 24/Norin 8	O
84	Aichinokaori	Improved	Hatsushimo/Mineasahi	O
85	Matsuribare	Improved	Aichi 56A/Mineasahi	O
86	Asanohikari	Improved	Koganebare//Aoisora/Hokuriku 103	O
87	Tsukinohikari	Improved	Koganebare//Aoisora/Hokuriku 103	O
88	Daichinokaze	Improved	Matsuribare/////Aoinokaze////Tsukinohikari///Aichi 77//Aichi 80/Akanezora	O
89	Nourin29	Improved	Norin 8/Norin 6	O
90	Asahi(旭)	*Landrace*		O
91	Kameji	*Landrace*		O
92	Shinriki	*Landrace*		O
93	Takenari	*Landrace*		O
94	Ginbozu	*Landrace*	A variety from Aikoku	O
95	Mineharuka	Improved	Chubu100//Chiyonishiki/Un1425	O
96	Akebono	Improved	Norin 12/Asahi	O
97	Yamabiko	Improved	Chukyoasahi/Norin 22	O
98	Nakate-shinsenbon	Improved	Norin 22/Hayabusa	O
99	Kinmaze	Improved	Ryosaku/Aichinakateasahi	O
100	Yamadanishiki	Improved	Yamadaho/Tankanwataribune	O
101	Norin22	Improved	Norin 8/Norin 6	O
102	Asahi(朝日)	*Landrace*		O
103	Hoshiaoba	Improved	Chugoku113/Ochikara	O
104	Kusanohoshi	Improved	Tashukei175/Akenohoshi	O
105	Tachisuzuka	Improved	Kusanohoshi//Aoinokaze/Chugoku156	O
106	Tachiayaka	Improved	Hoshiaoba/(Aoinokaze/Chugoku156)//Hoshiaoba///Hoshiaoba	O
107	Koinoyokan	Improved	Kinumusume/Chugoku178	
108	Himegonomi	Improved	Milkyqueen/Chugoku169	
109	Haigokoro	Improved	Milkyprincess/Kyo5-7	
110	Oidemai	Improved	AwaminoriHohoemi	
111	Reiho	Improved	Houyoku/Ayanishiki	O
112	Hiyokumochi	Improved	Houyoku/Iwaimochi	O
113	Houyoku	Improved	Jukkoku/Zensho 26	O
114	Jukkoku	*Landrace*		O
115	Nourin18	Improved	Oitamii 120/Takara	O
116	Hinohikari	Improved	Koganebare/Koshihikari	O
117	Nishihomare	Improved	Toyotama/Chugoku 45	O
118	Koganemasari	Improved	Nipponbare/Koganenishiki	O
119	Yumehikari	Improved	Kinuhikari/Koshihikari	O
120	Omachi	*Landrace*		O
121	Shirosenbon	*Landrace*		O
122	Mizuhochikara	Improved	Ouu326/86SH283	O
123	Tachiaoba	Improved	Hakei906/(42-1-1)F2/95SH50	O
124	Kinumusume	Improved	Kinuhikari/Matsuribare	O
125	Nikomaru	Improved	Kinumusume/Hokuriku174	O
126	Tachiharuka	Improved	Tachiaoba/Mineharuka	O
127	Mizuho	Improved	Miyakei1430/Suzukaze	O
128	Minaminishiki	Improved	Hokuriku174/Chubu98	O
