# Supplementary material for: Description of grain weight distribution leading to genomic selection for grain-filling characteristics in rice
Source: PLoS One. 2018 Nov 20;13(11):e0207627. doi: 10.1371/journal.pone.0207627 (PMC6245794; doi:10.1371/journal.pone.0207627)
Supplement: S1 Fig — Blue and green points represent the weight of inferior and superior grain derived from the center primary branch on each panicle. (PDF) [file pone.0207627.s002.pdf]

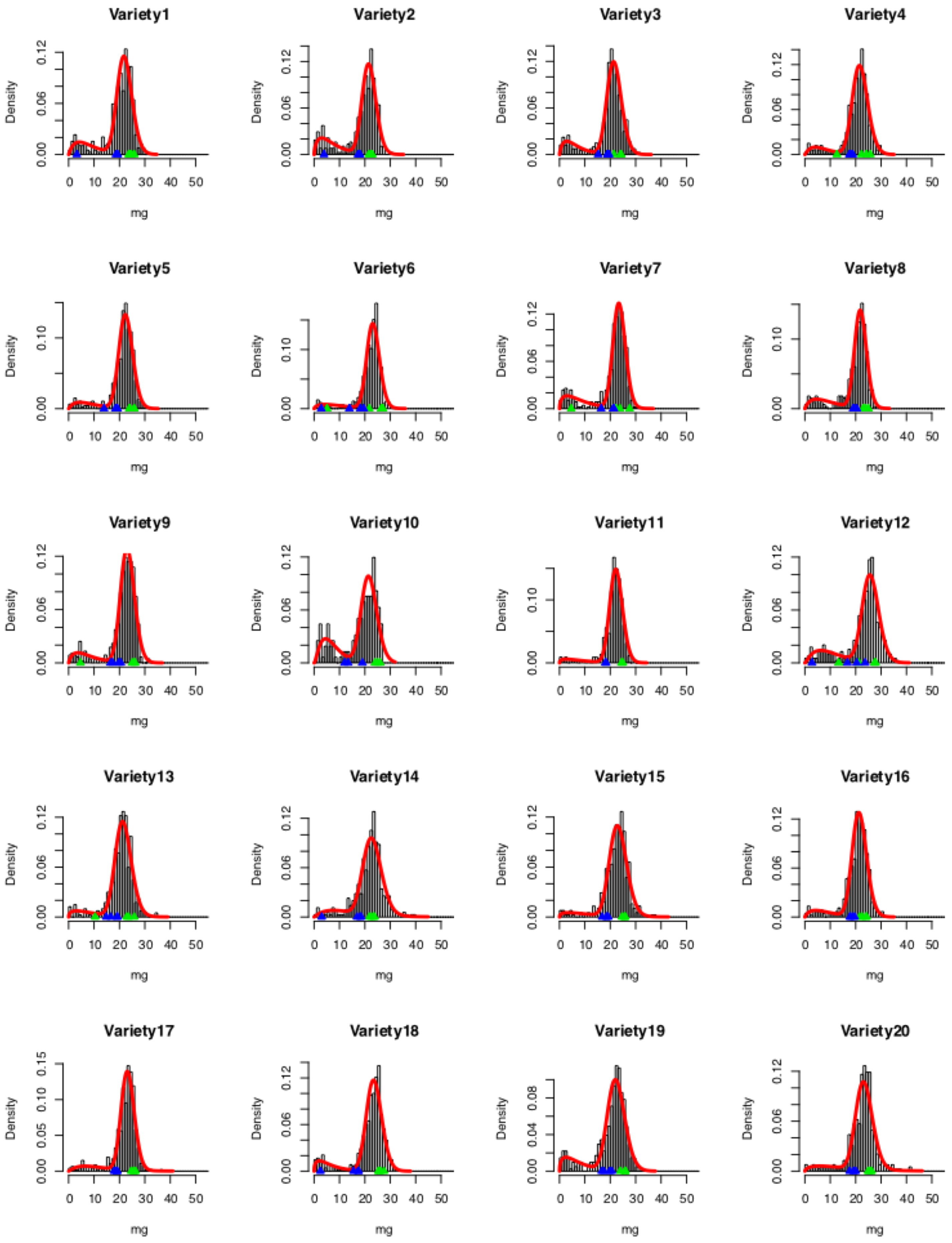

Supplementary Figure S1 Histogram and estimated probability density function (red line) of grain weight. Blue and green points represent the weight of inferior and superior grain derived from the center primary branch on each panicle.

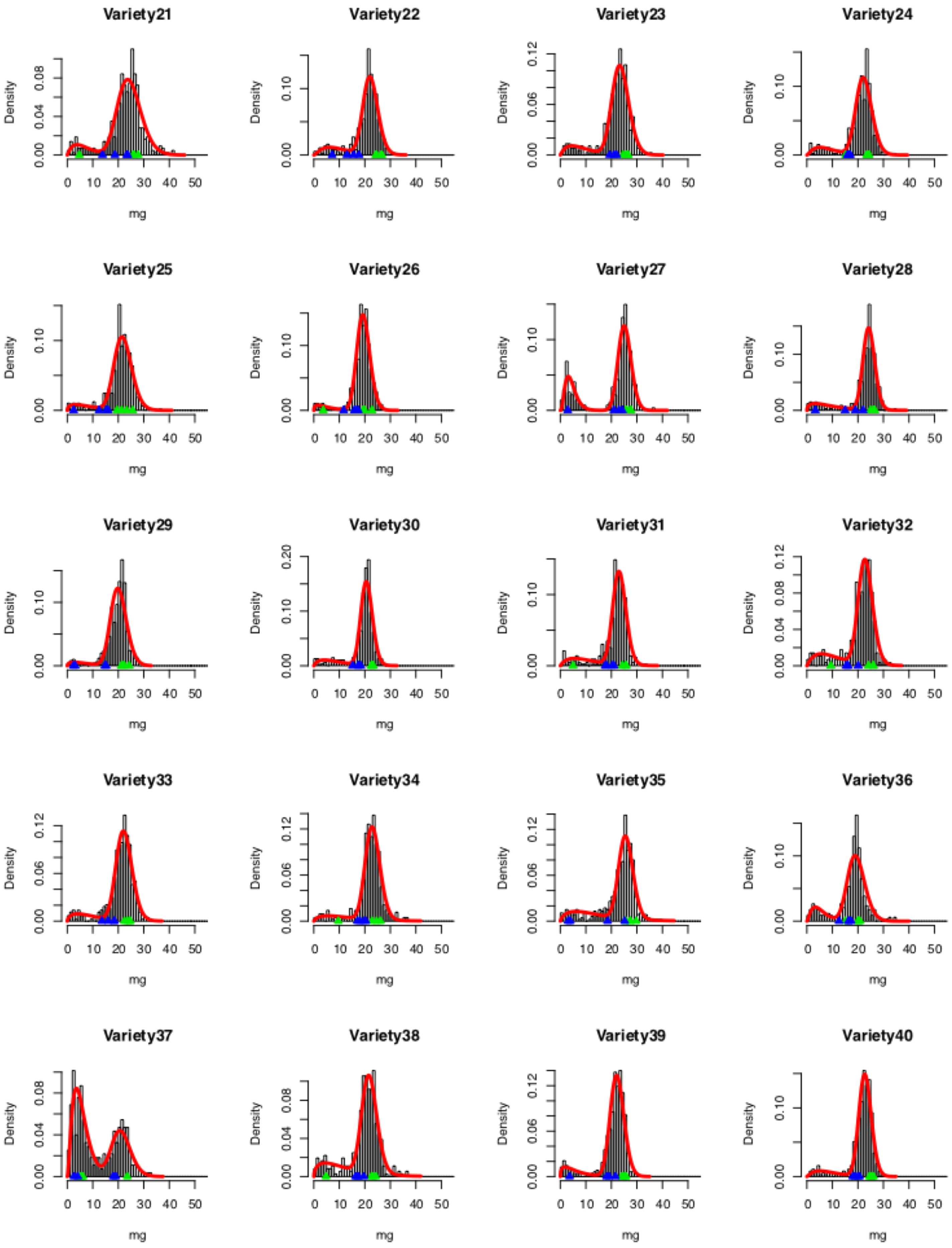

Supplementary Figure S1 (Continued)

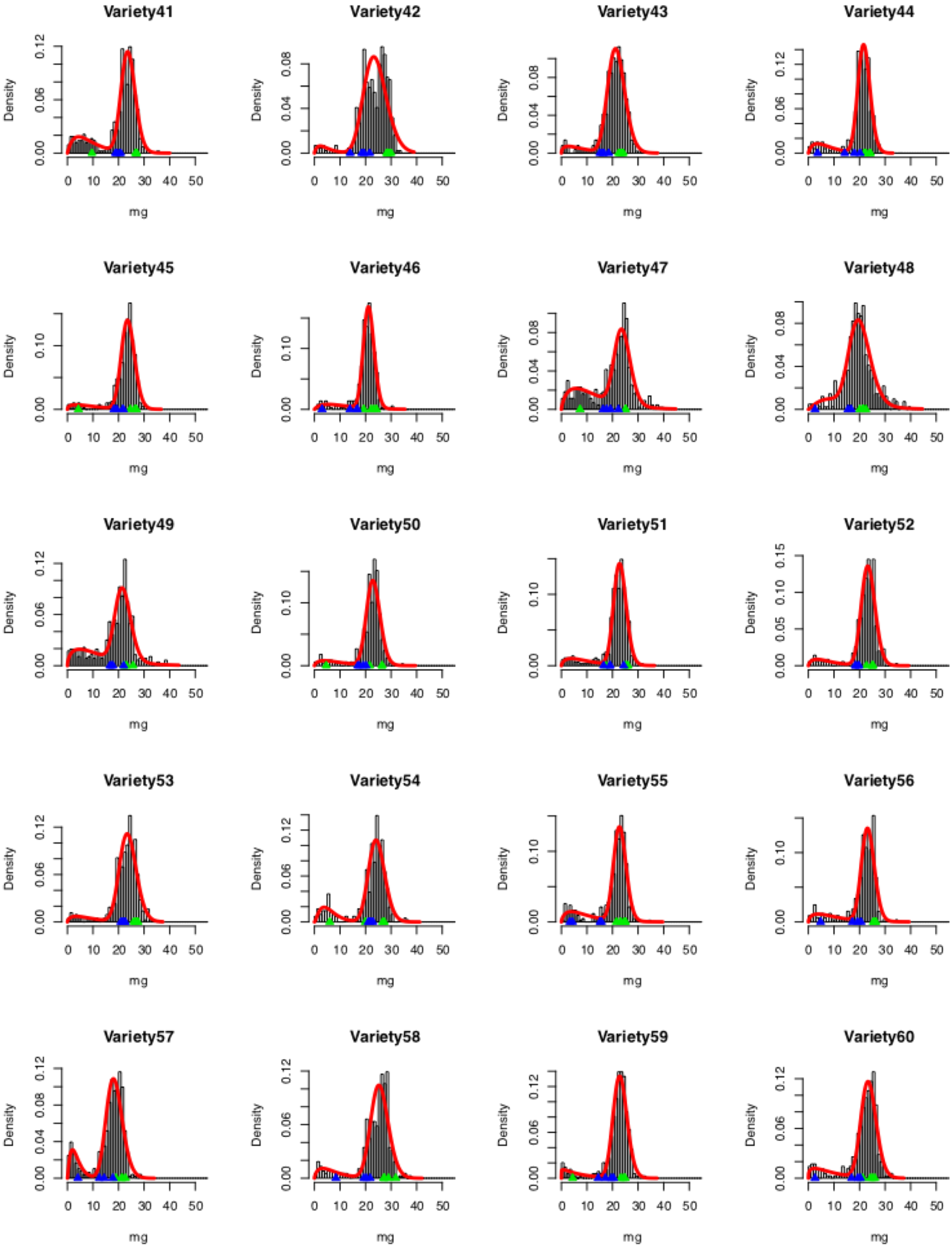

Supplementary Figure S1 (Continued)

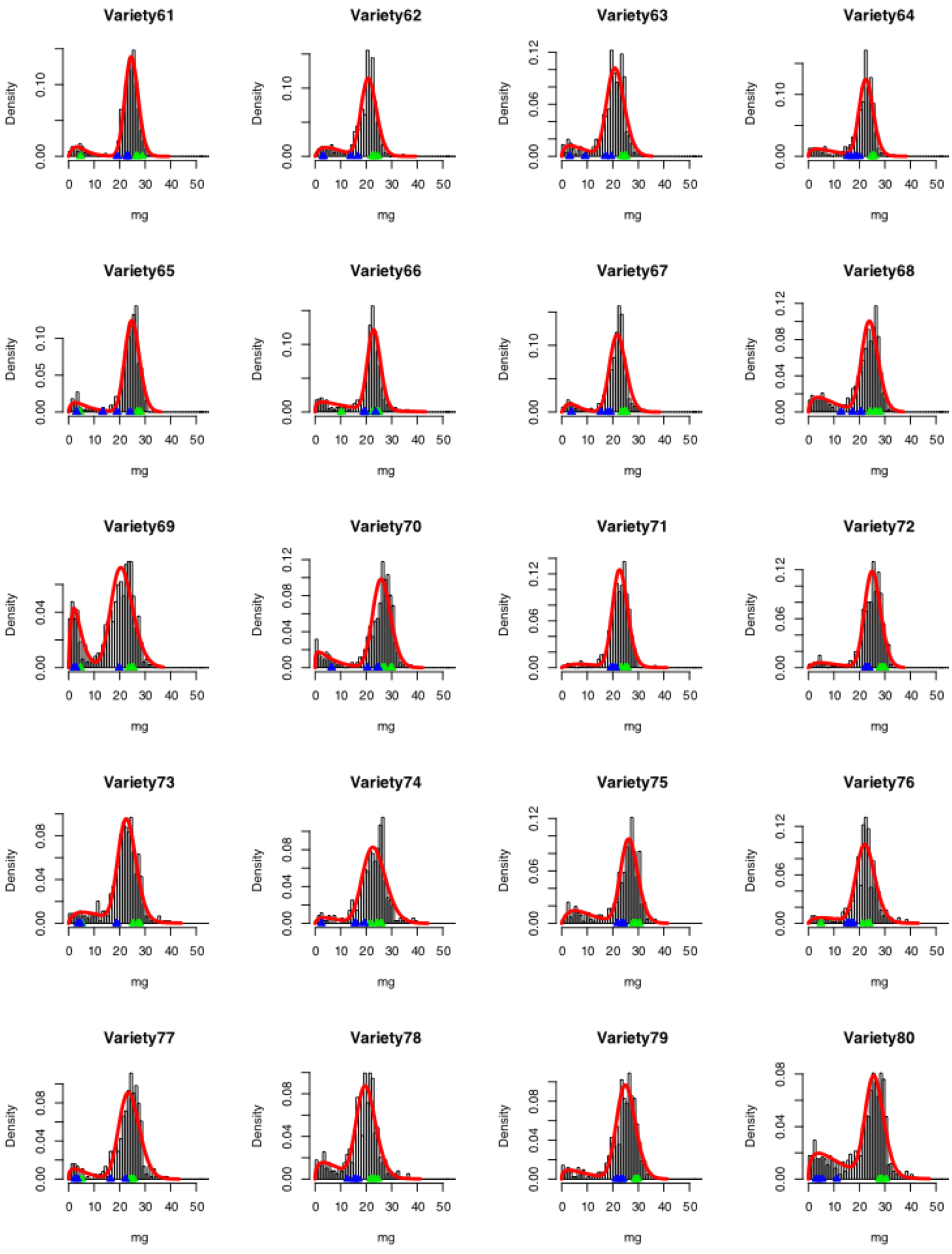

Supplementary Figure S1 (Continued)

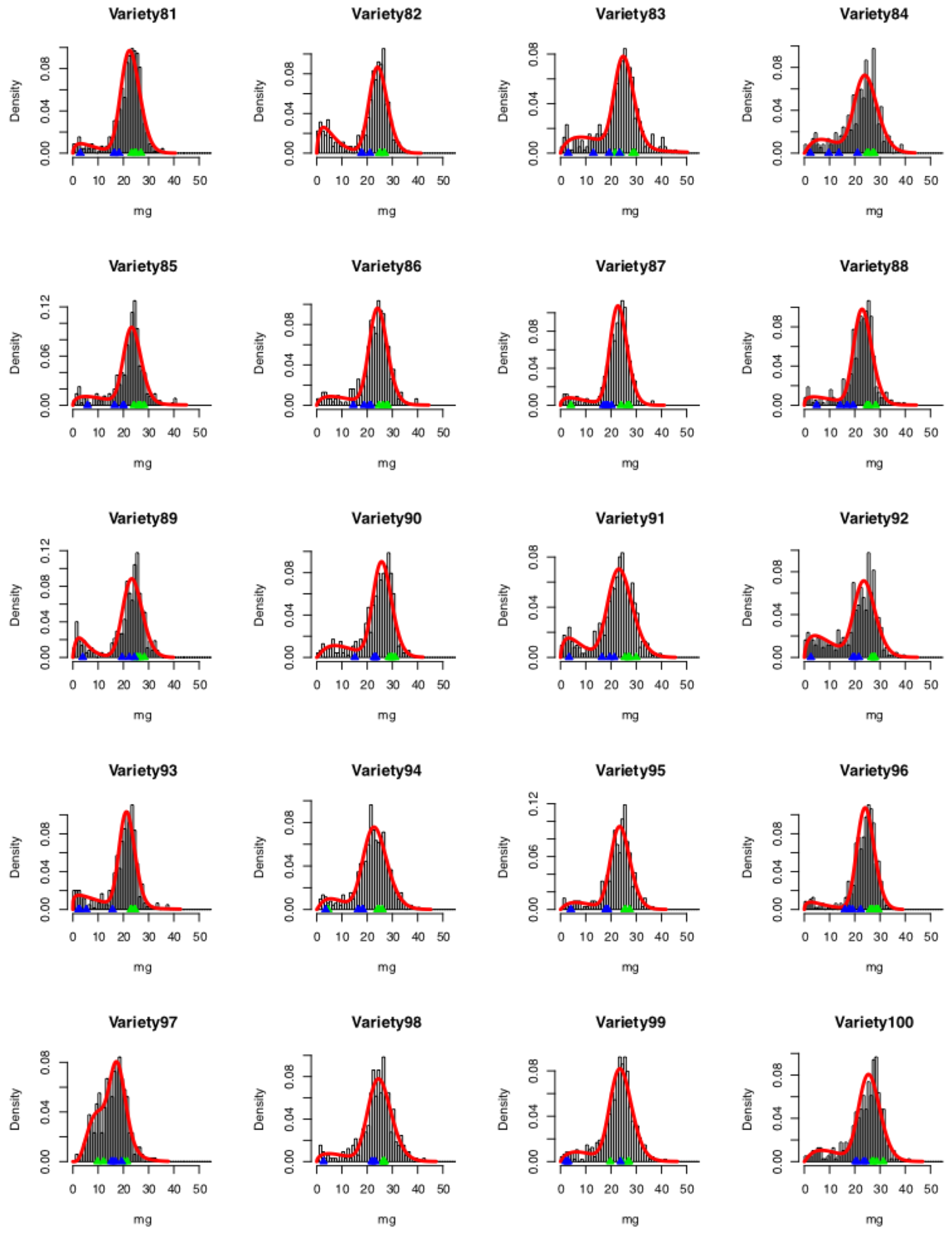

Supplementary Figure S1 (Continued)

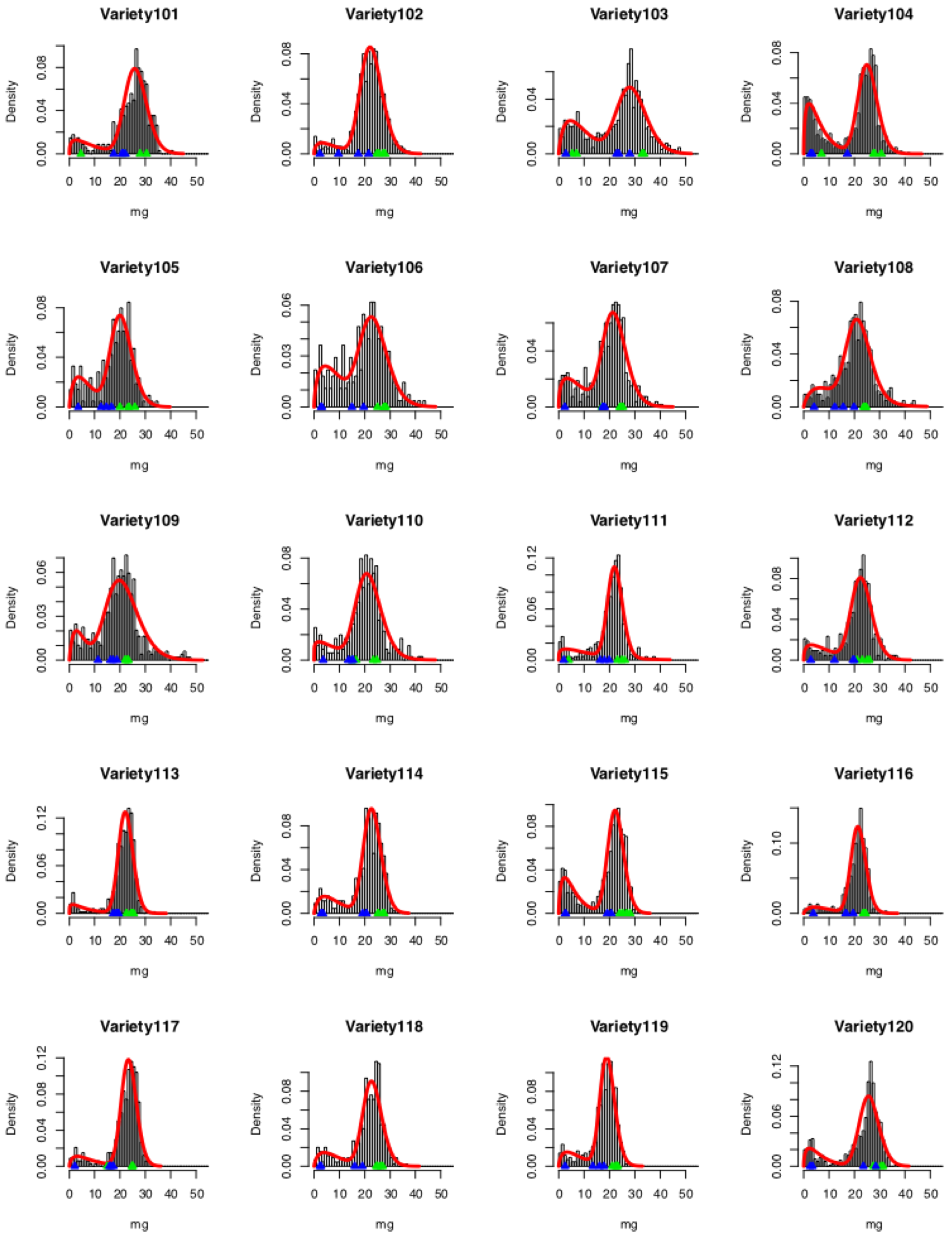

Supplementary Figure S1 (Continued)

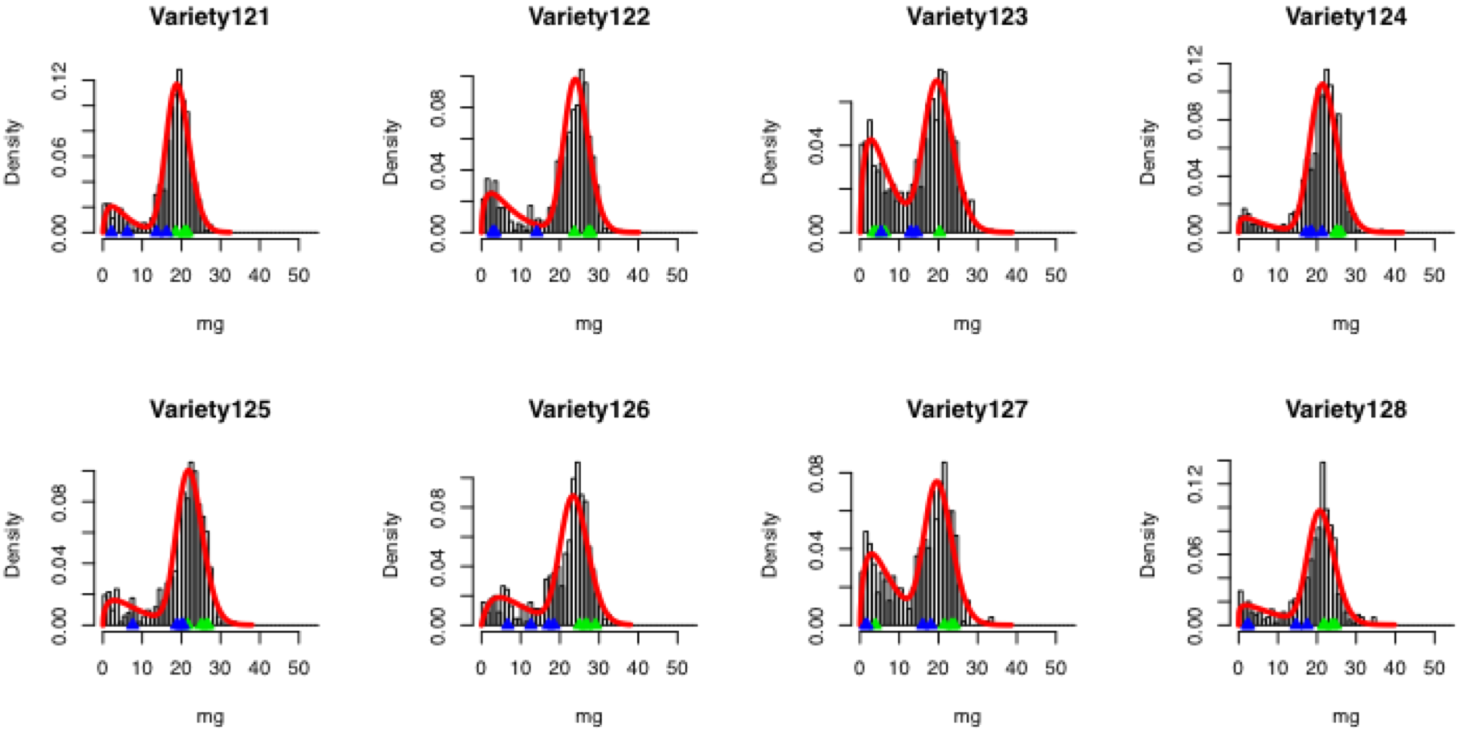

Supplementary Figure S1 (Continued)
