# Supplementary material for: Description of grain weight distribution leading to genomic selection for grain-filling characteristics in rice
Source: PLoS One. 2018 Nov 20;13(11):e0207627. doi: 10.1371/journal.pone.0207627 (PMC6245794; doi:10.1371/journal.pone.0207627)
Supplement: S2 Fig — (PDF) [file pone.0207627.s003.pdf]

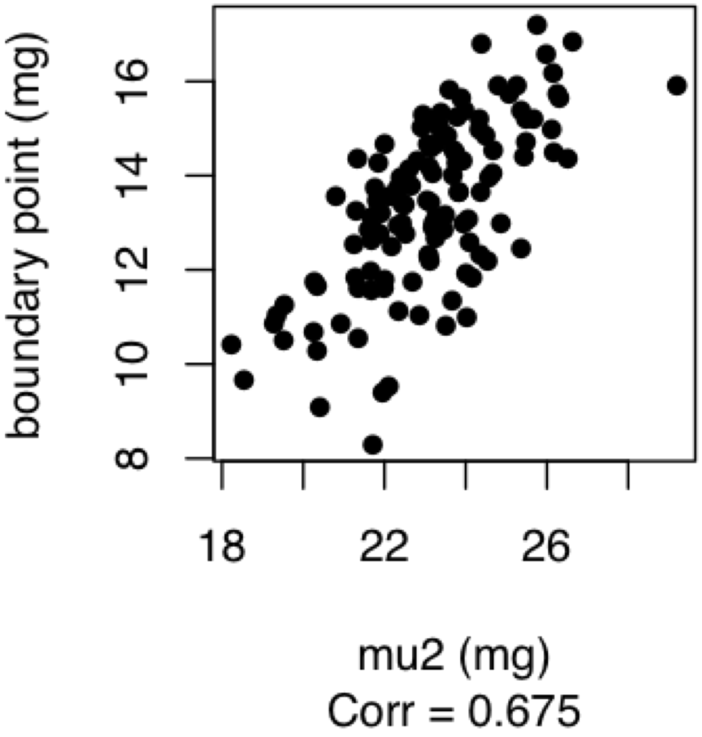

Supplementary Figure S2 Relation between the grain weight at the boundary point in the grain weight distribution and the distribution parameter mu2.
