# Supplementary material for: Description of grain weight distribution leading to genomic selection for grain-filling characteristics in rice
Source: PLoS One. 2018 Nov 20;13(11):e0207627. doi: 10.1371/journal.pone.0207627 (PMC6245794; doi:10.1371/journal.pone.0207627)
Supplement: S3 Fig — Principal component analysis of grain weight distribution parameters among 128 cultivars (a) and representative grain weight distribution of each cultivar group (b). Cultivars were grouped by the k-means method using grain weight distribution parameters. Each color represents each cultivar group, and the same color is used for a specific cultivar group both in (a) and (b). Point size in (a) represents the sink-filling rate in each cultivar. In (b), the red solid line shows the distribution based on the median of each parameter among all cultivars. The dashed lines show the distribution using the median of each parameter in each cultivar group. (PDF) [file pone.0207627.s004.pdf]

(a)

(b)

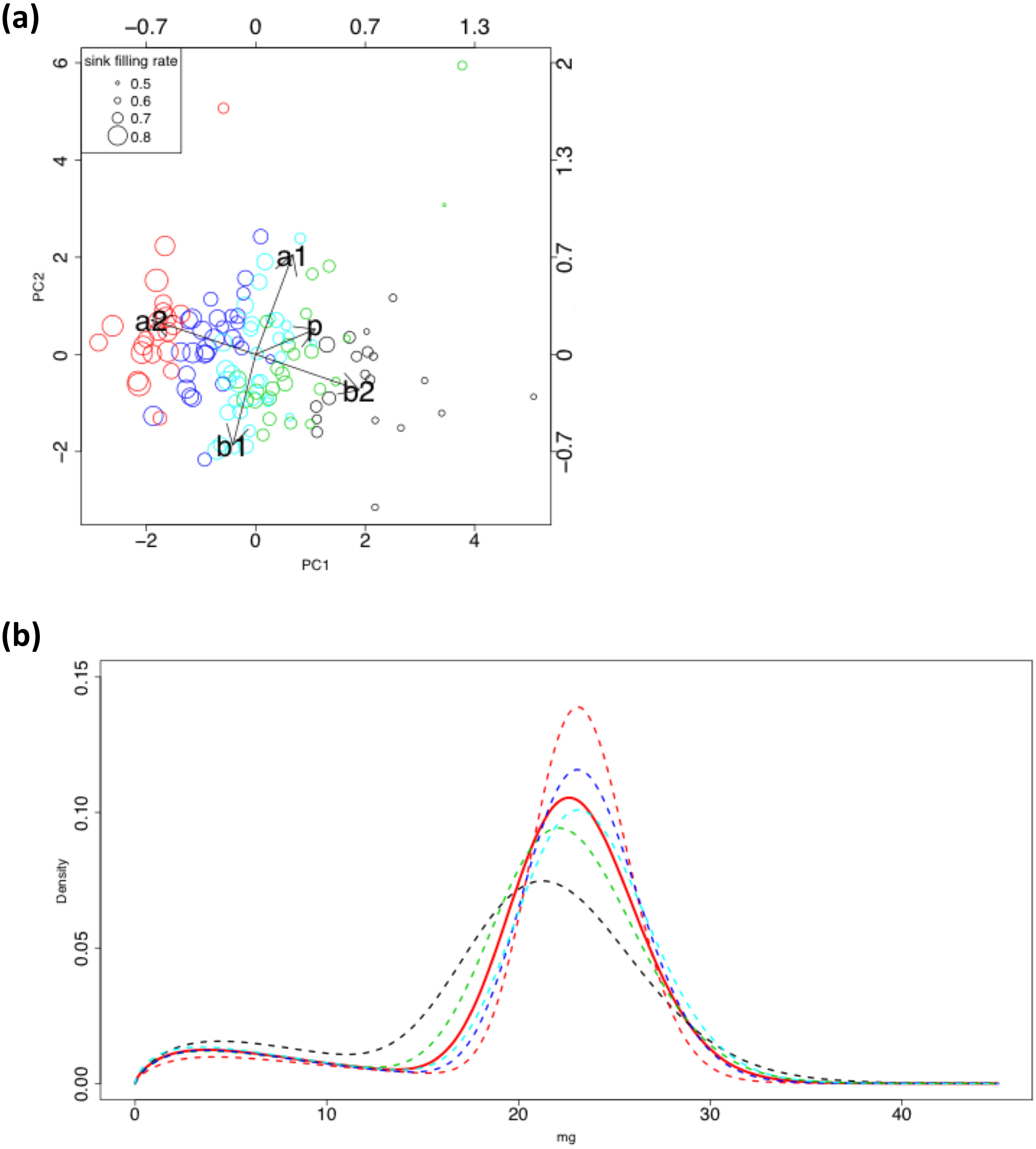

Supplementary Figure S3 Principal component analysis of grain weight distribution parameters among 128 cultivars (a) and representative grain weight distribution of each cultivar group (b). Cultivars were grouped by the k-means method using grain weight distribution parameters. Each color represents each cultivar group, and the same color is used for a specific cultivar group both in (a) and (b) . Point size in (a) represents the sink-filling rate in each cultivar. In (b), the red solid line shows the distribution based on the median of each parameter among all cultivars. The dashed lines show the distribution using the median of each parameter in each cultivar group.
