# Supplementary material for: Description of grain weight distribution leading to genomic selection for grain-filling characteristics in rice
Source: PLoS One. 2018 Nov 20;13(11):e0207627. doi: 10.1371/journal.pone.0207627 (PMC6245794; doi:10.1371/journal.pone.0207627)
Supplement: S4 Fig — Probability density function (left) and cumulative probability (right) of observed and predicted grain weight distribution. Left-side plot among two plots with the same Variety ID shows the result of comparison between the observed probability density function (red line) of grain weight and predicted ones (blue). Right-side plot shows the result of comparison between cumulative probability of observed and predicted grain weight distribution. These results were derived from genomic prediction for grain weight distribution parameters by GBLUP (solid line), PLS using 1 parameter-group (dashed line), and PLS using 3 parameter-group (dotted line). (PDF) [file pone.0207627.s005.pdf]

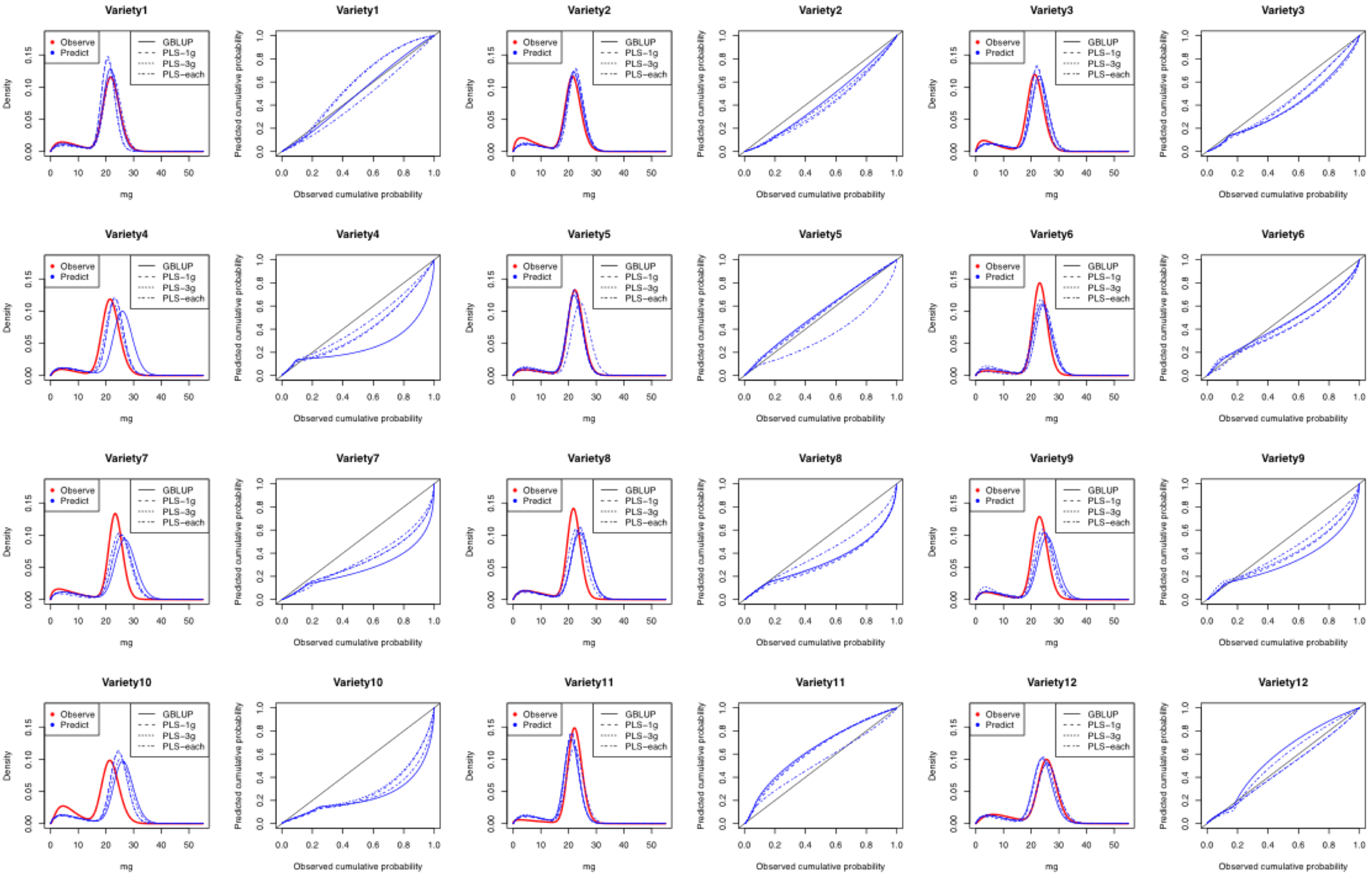

Supplementary Figure S4 Probability density function (left) and cumulative probability (right) of observed and predicted grain weight distribution. Left-side plot among two plots with the same Variety ID shows the result of comparison between the observed probability density function (red line) of grain weight and predicted ones (blue). Right-side plot shows the result of comparison between cumulative probability of observed and predicted grain weight distribution. These results were derived from genomic prediction for grain weight distribution parameters by GBLUP (solid line), PLS using 1 parameter-group (dashed line), and PLS using 3 parameter-group (dotted line).

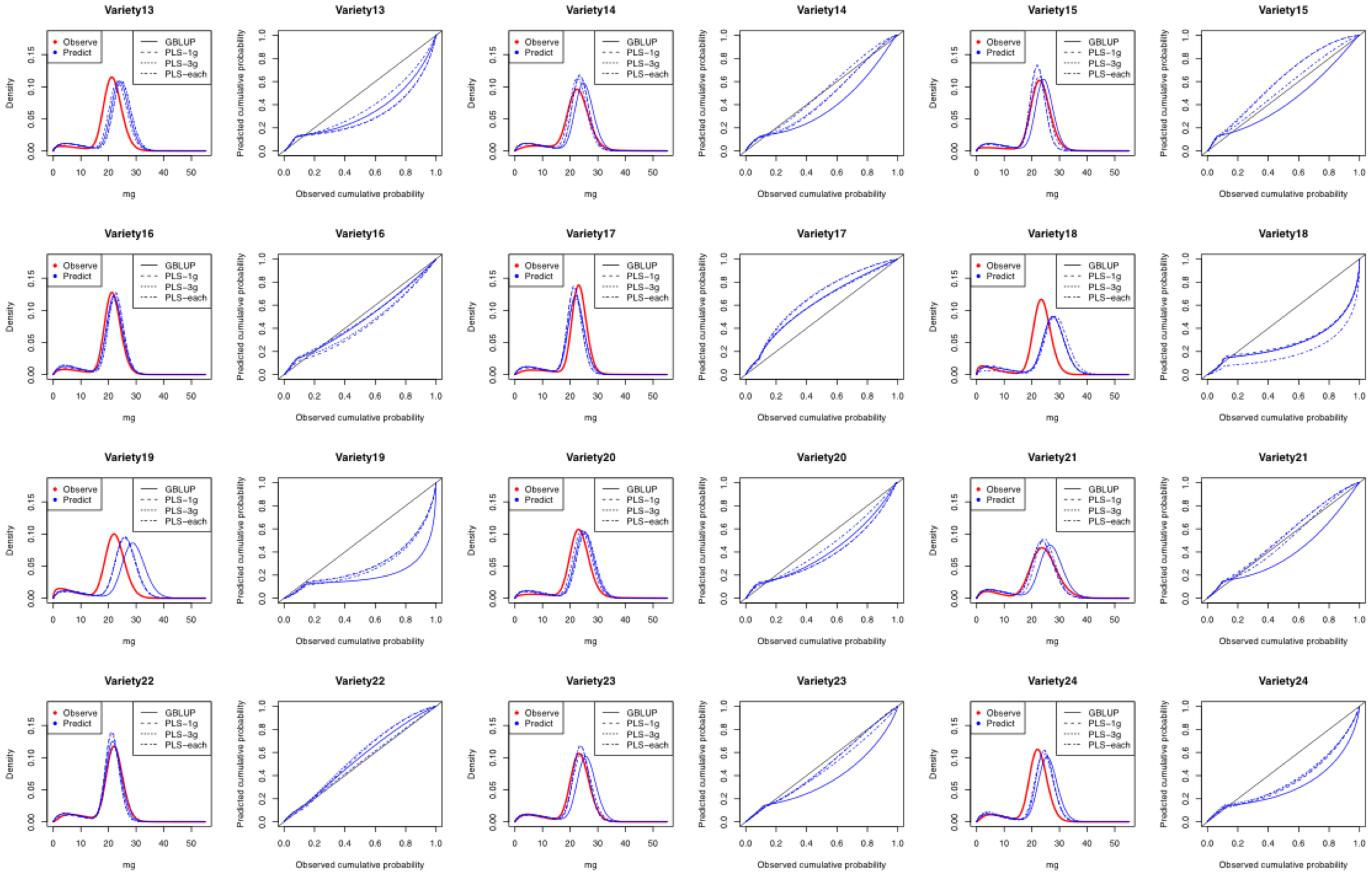

Supplementary Figure S4 (Continued)

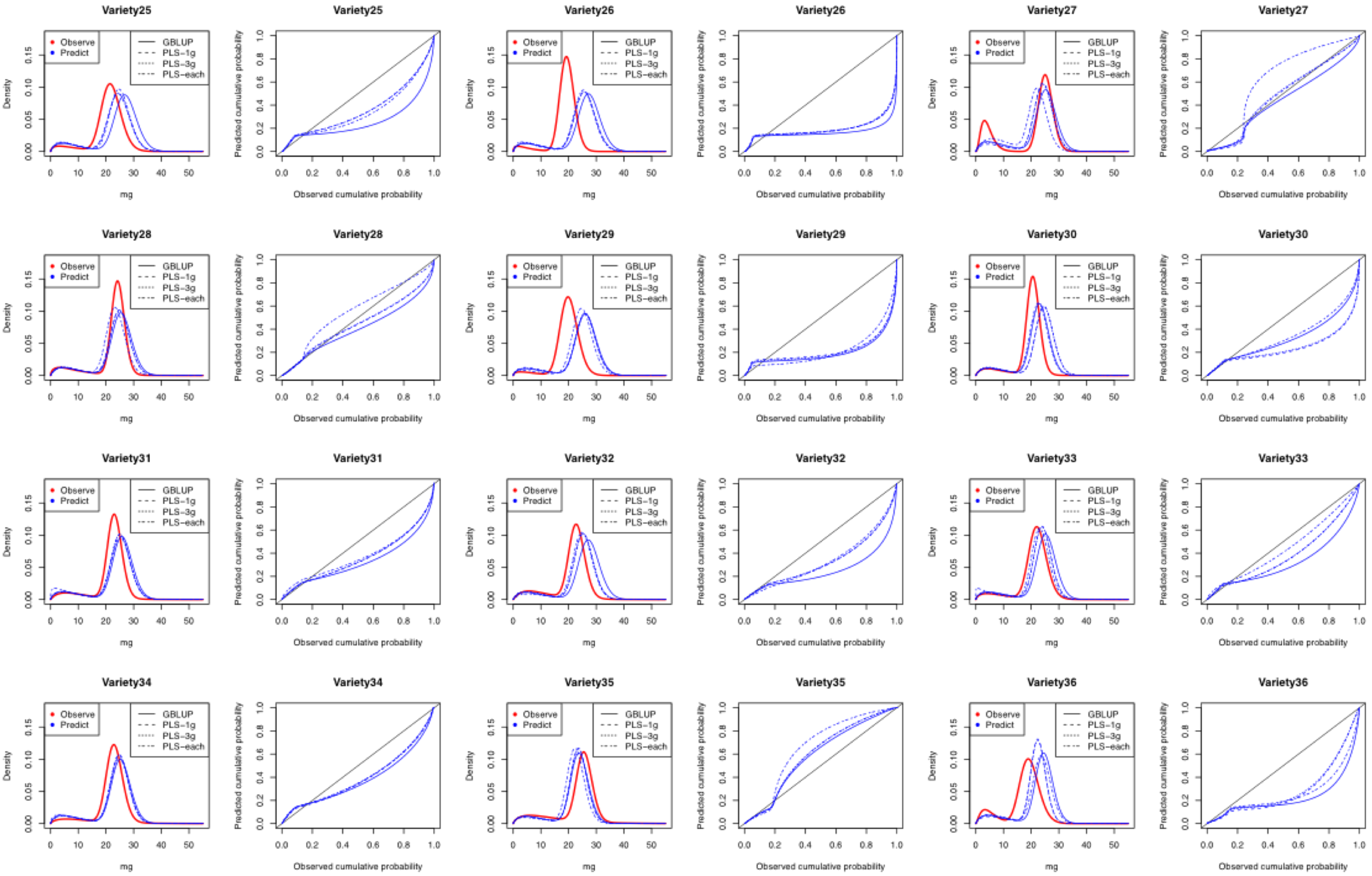

Supplementary Figure S4 (Continued)

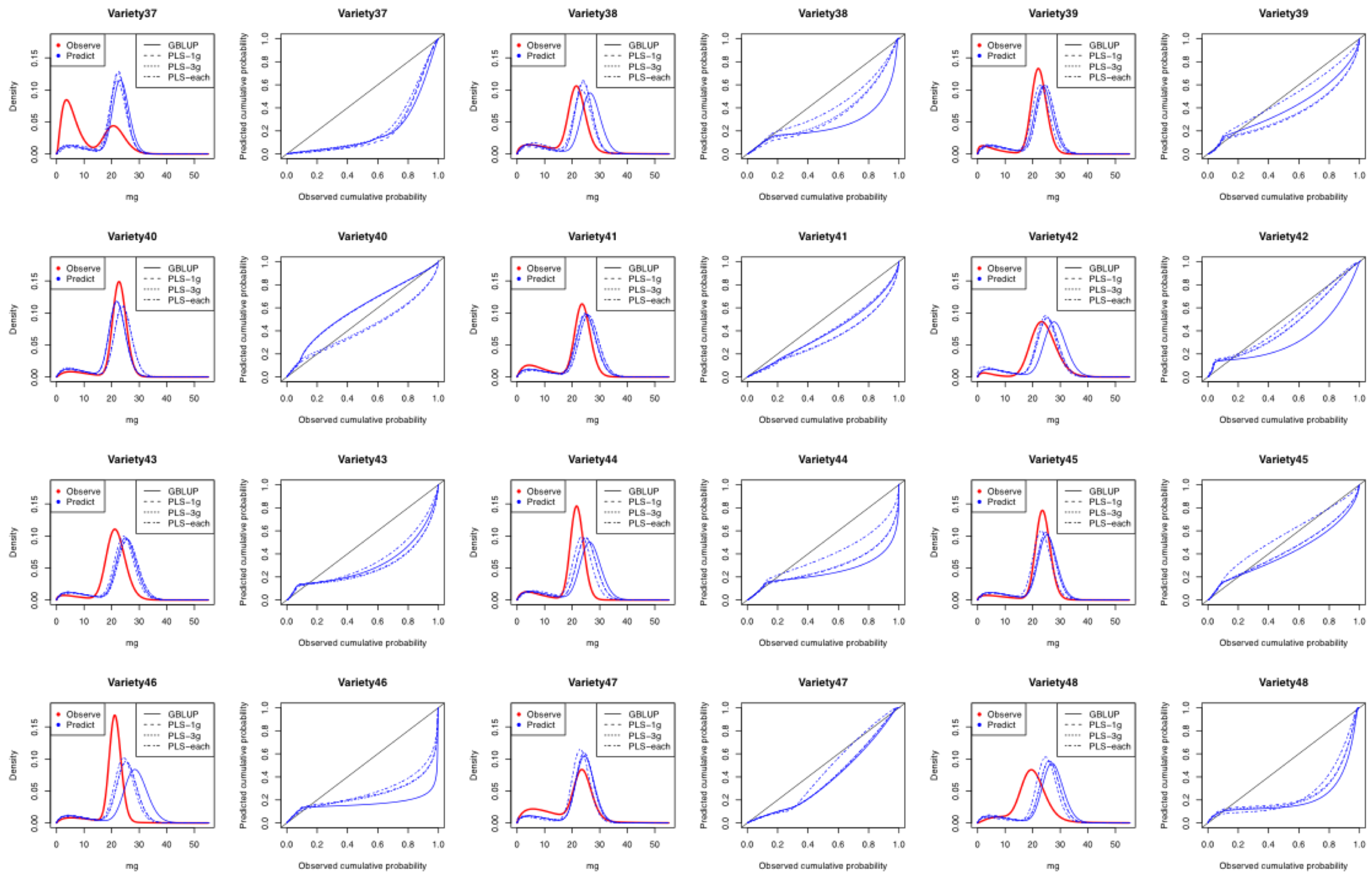

Supplementary Figure S4 (Continued)

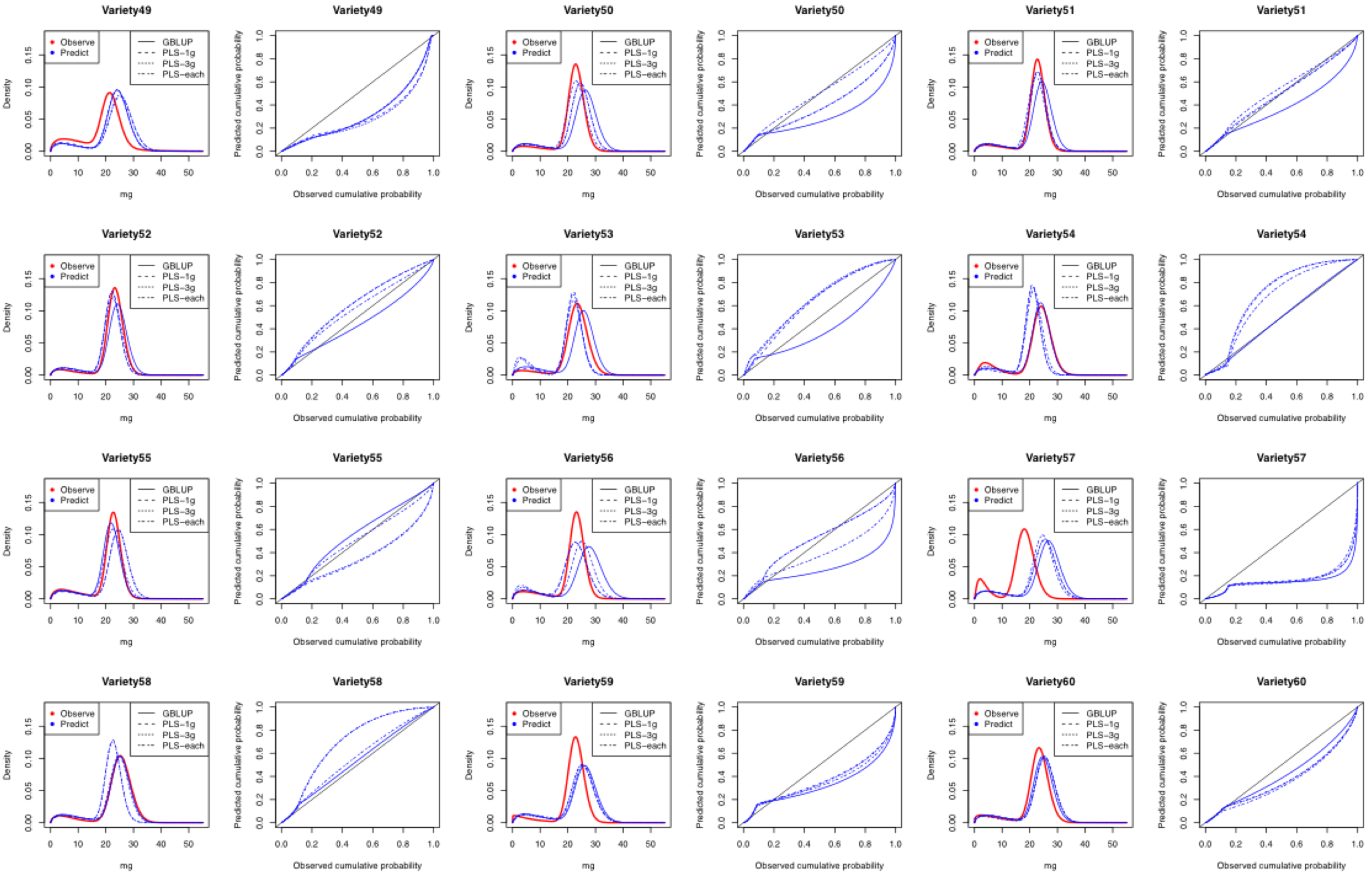

Supplementary Figure S4 (Continued)

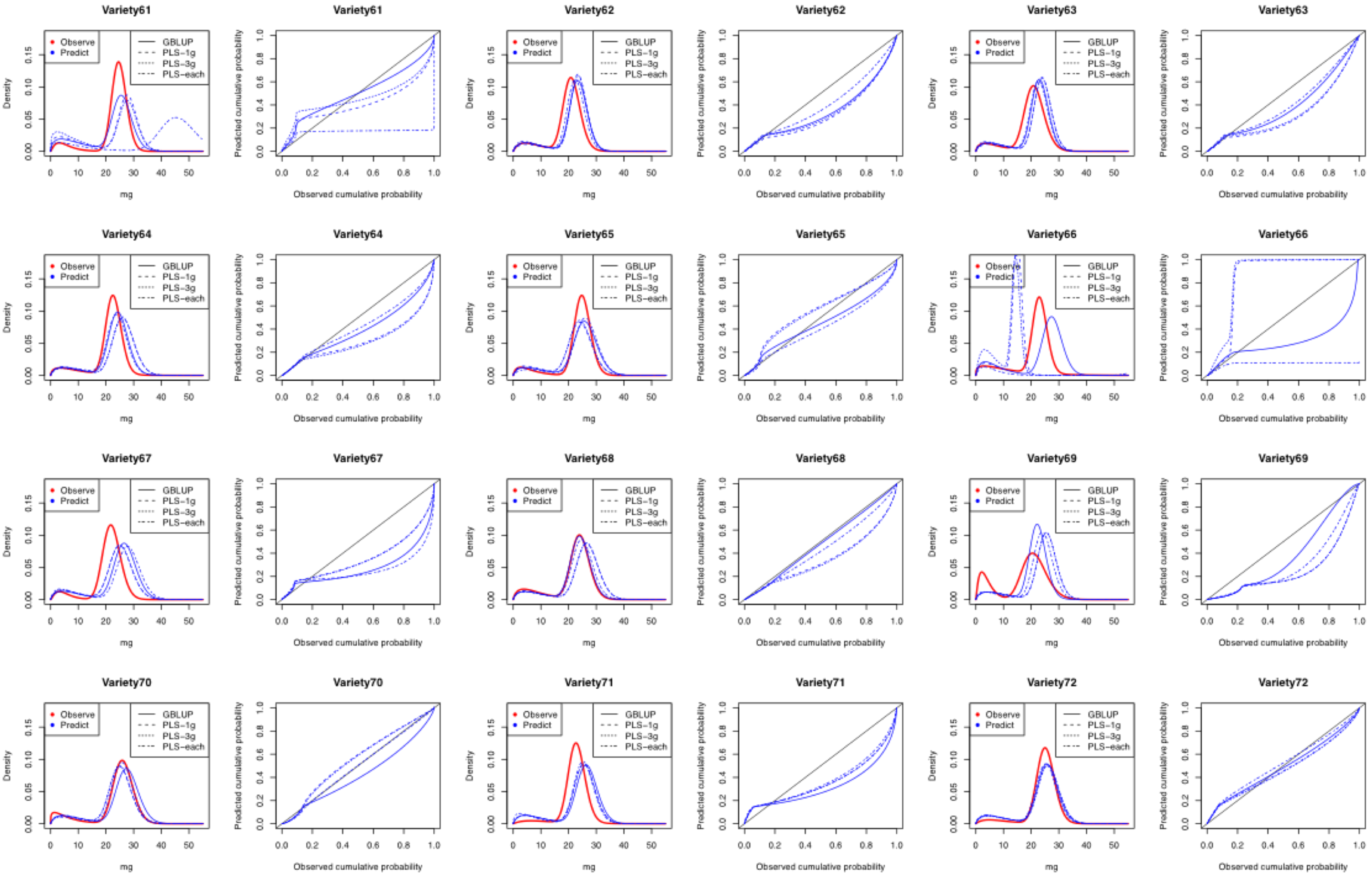

Supplementary Figure S4 (Continued)

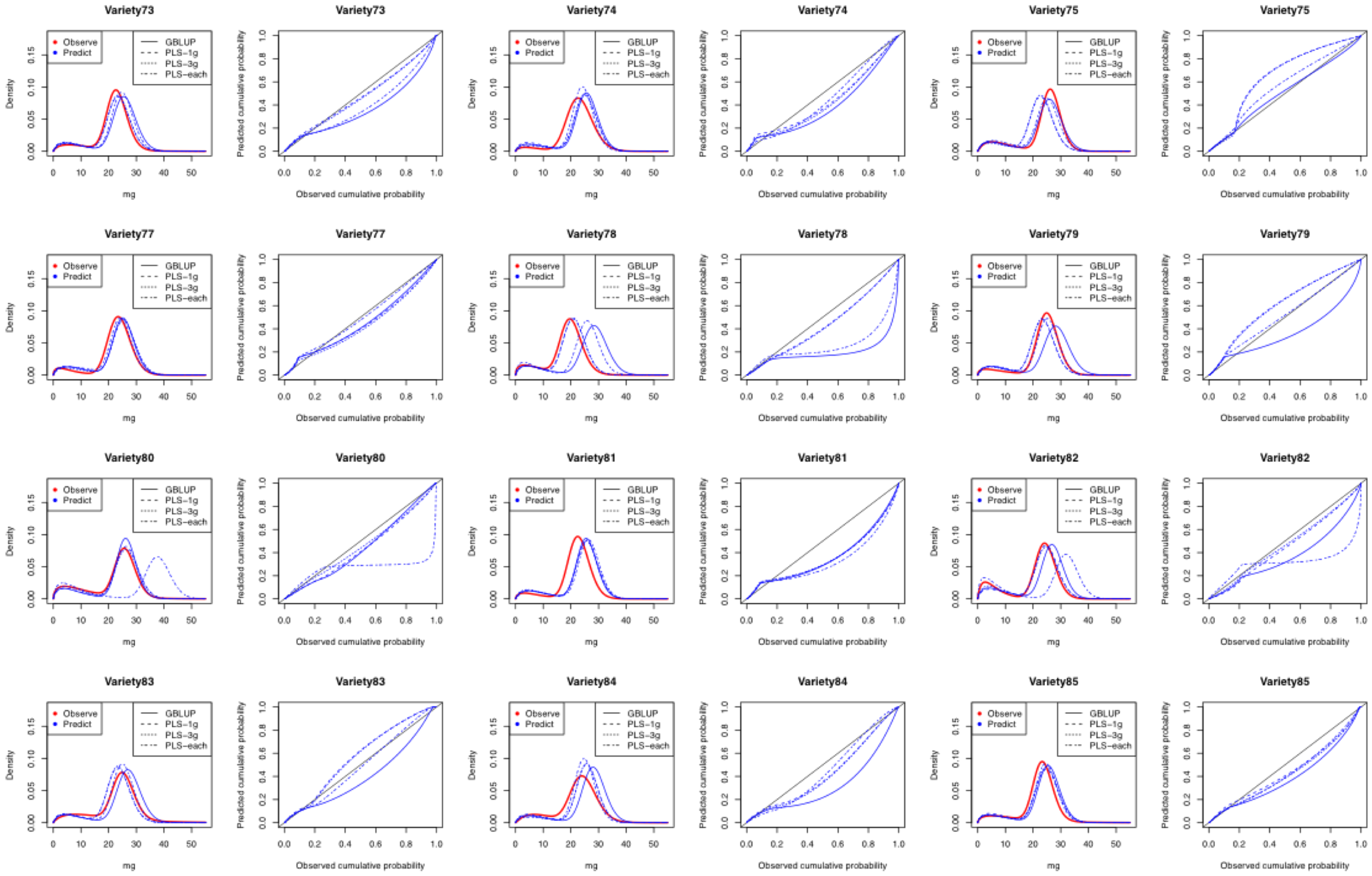

Supplementary Figure S4 (Continued)

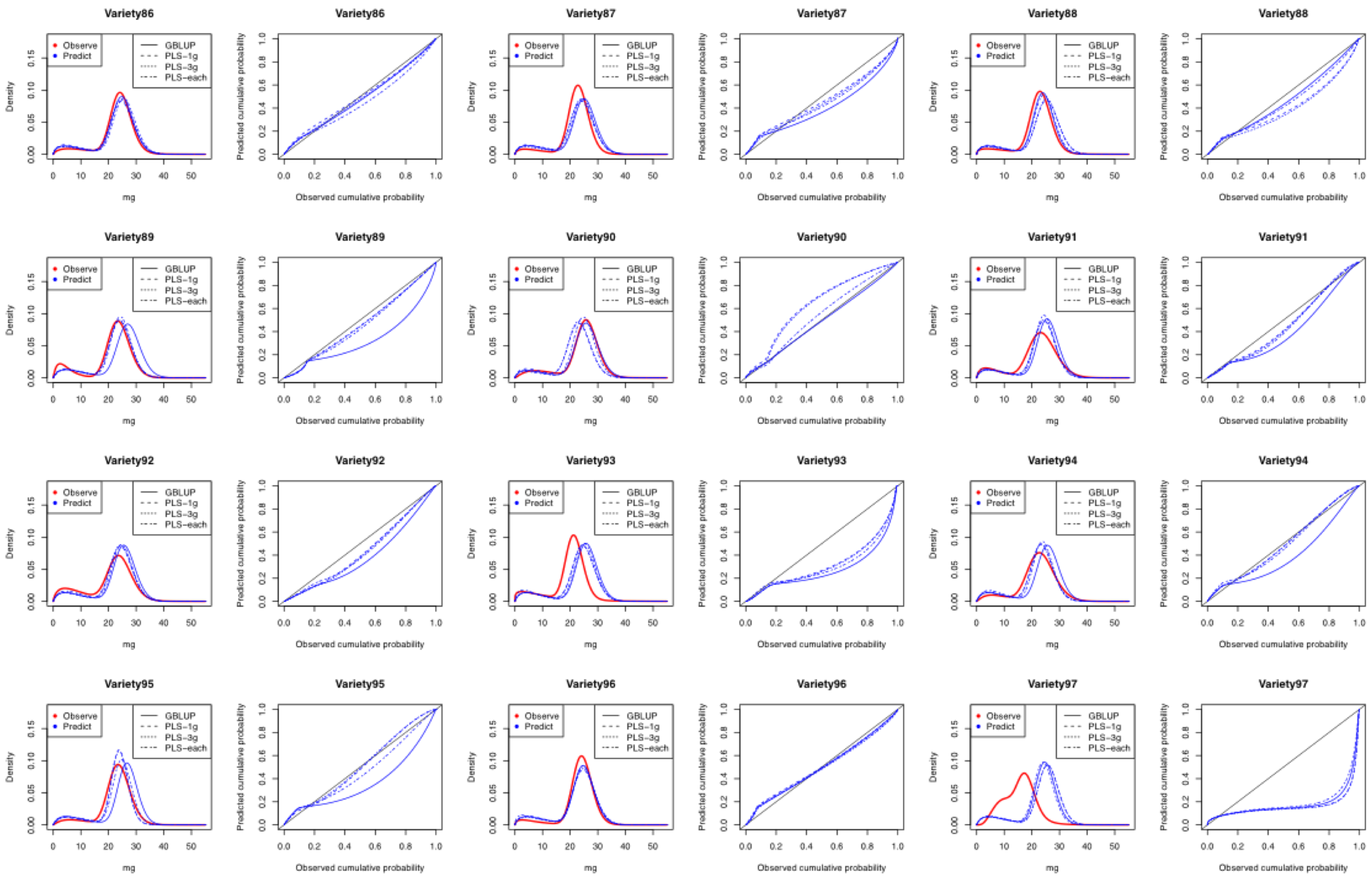

Supplementary Figure S4 (Continued)

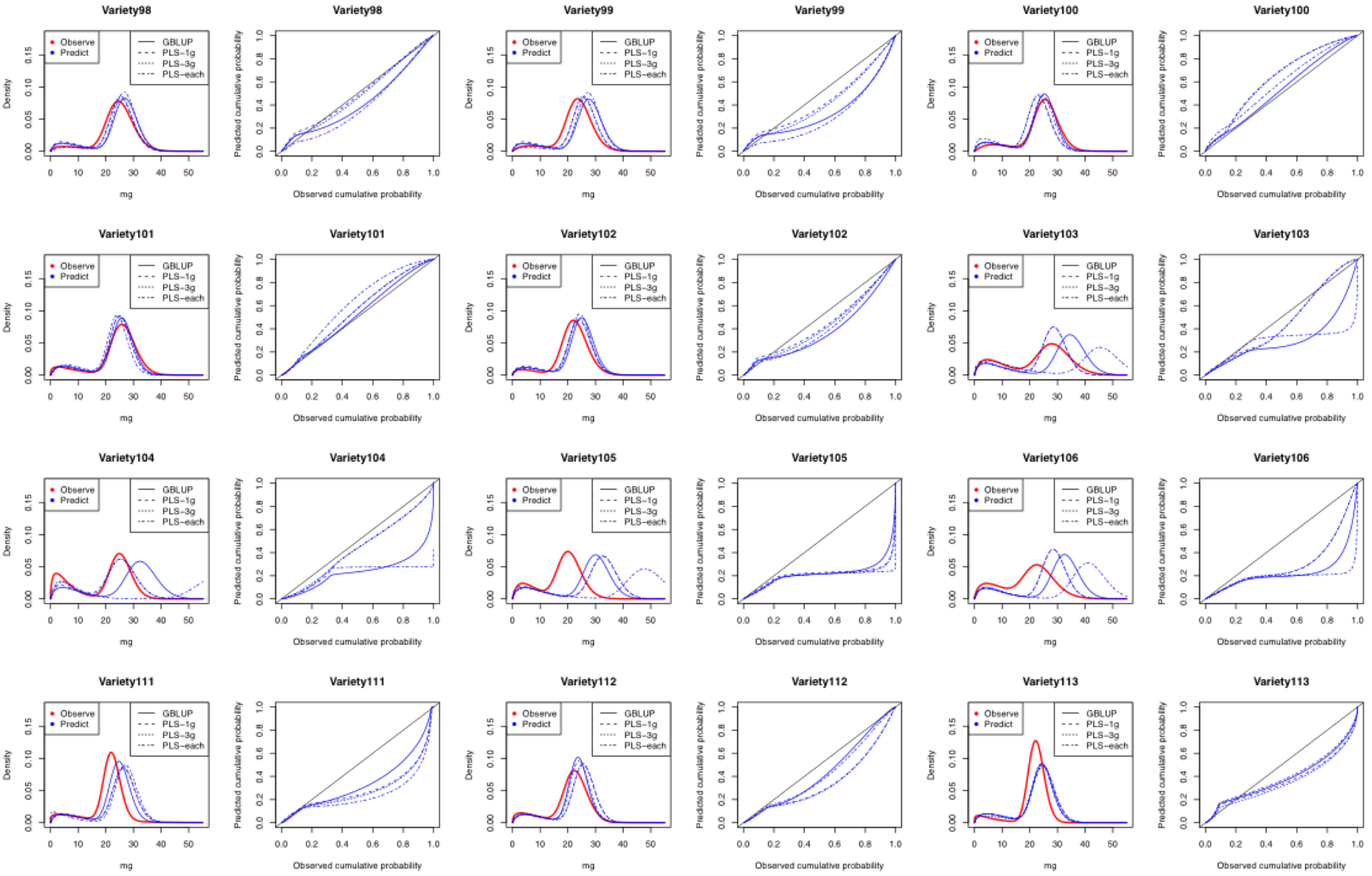

Supplementary Figure S4 (Continued)

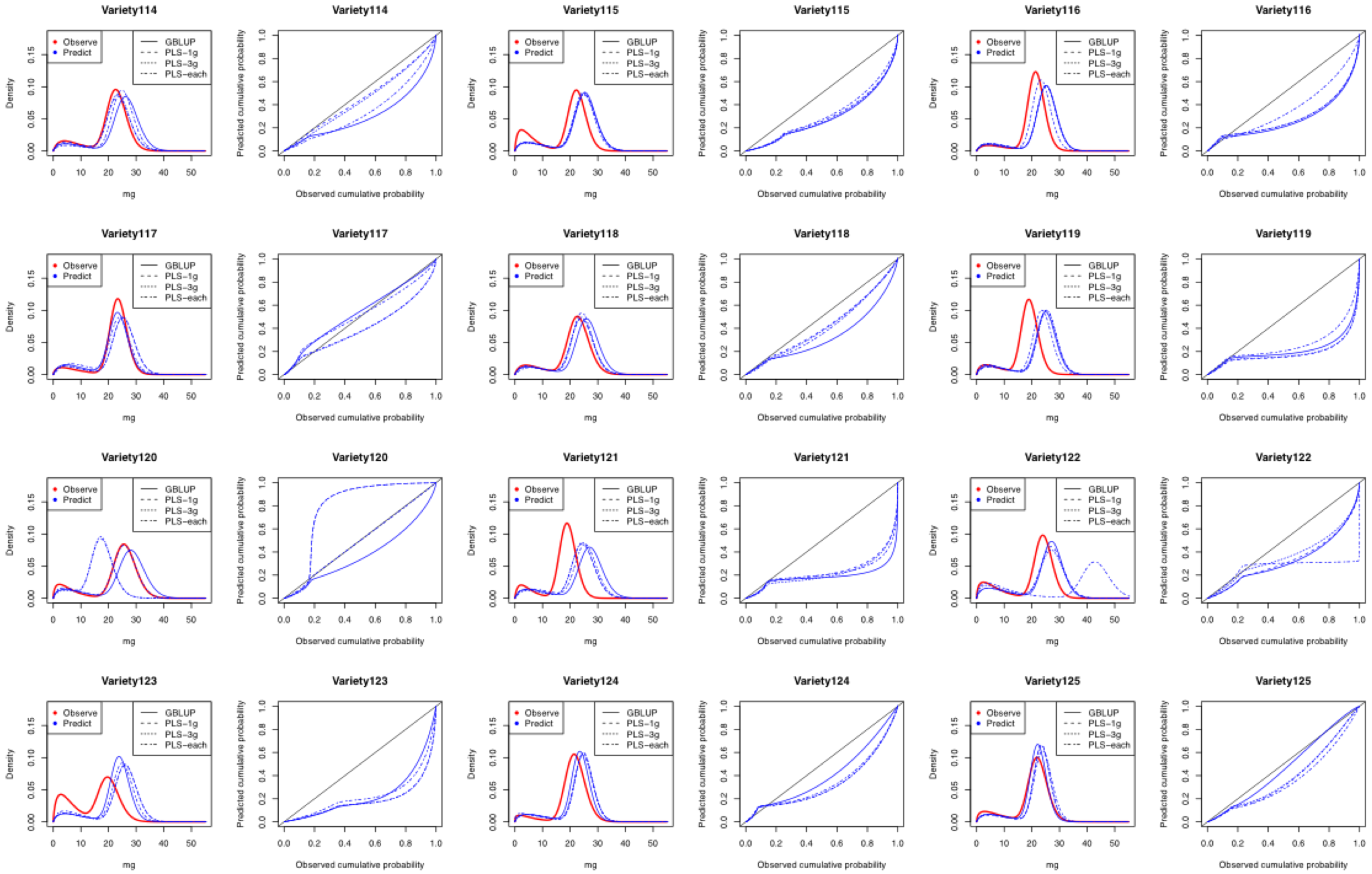

Supplementary Figure S4 (Continued)

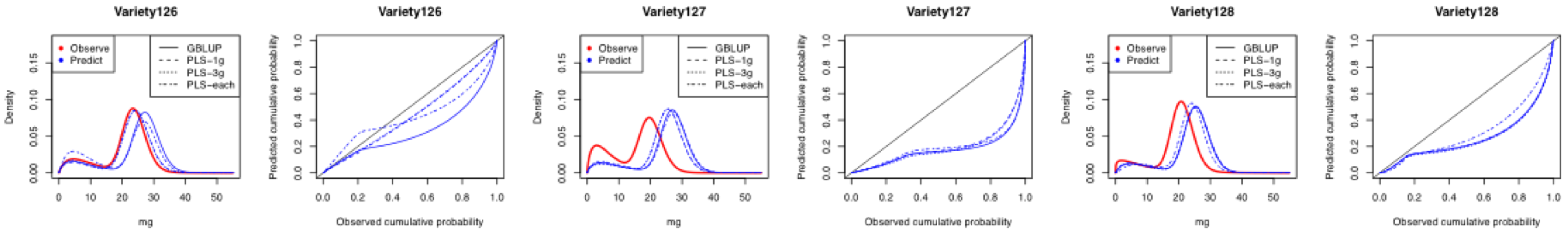

Supplementary Figure S4 (Continued)
