# Supplementary material for: Description of grain weight distribution leading to genomic selection for grain-filling characteristics in rice
Source: PLoS One. 2018 Nov 20;13(11):e0207627. doi: 10.1371/journal.pone.0207627 (PMC6245794; doi:10.1371/journal.pone.0207627)
Supplement: S5 Fig — (a) The X and Y-axes show the scores of PC1 and PC2, respectively. (b) The X and Y-axes show the scores of PC1 and PC3, respectively. The size of the points represents the predicted residual error in the prediction by GBLUP for each cultivar. (PDF) [file pone.0207627.s006.pdf]

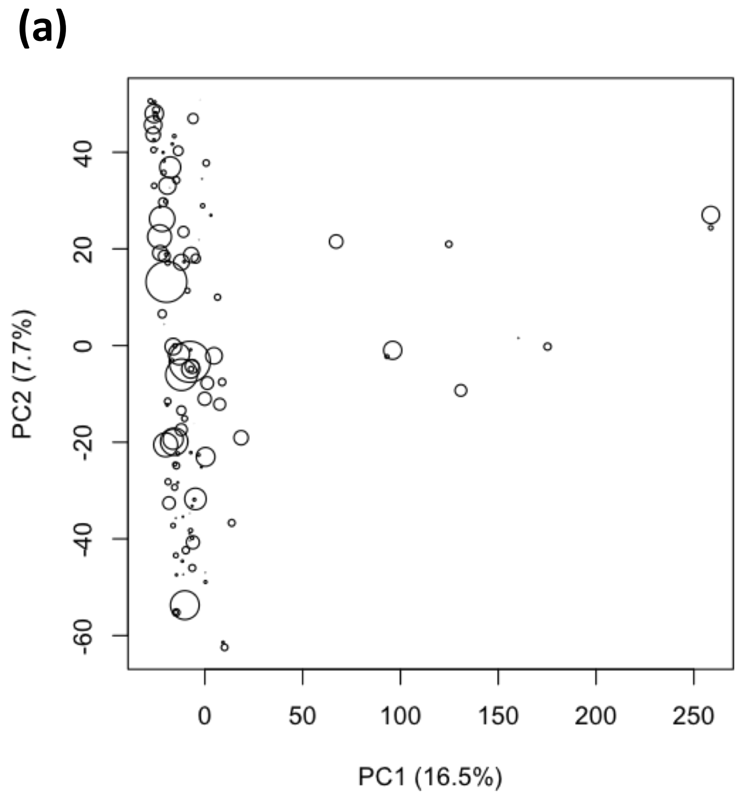

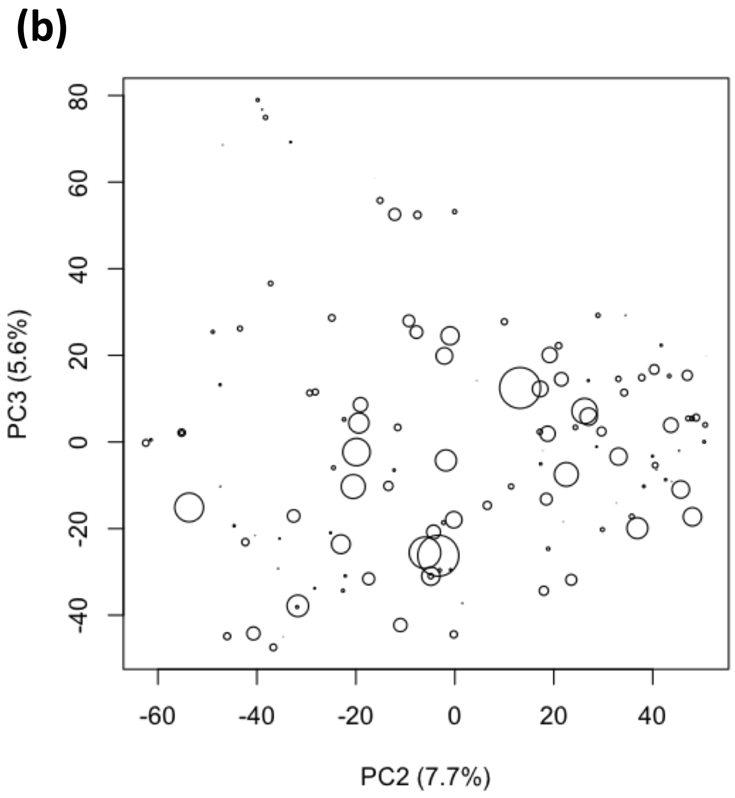

Supplementary Figure S5 Principal component analysis using genome-wide markers among 128 cultivars. (a) The X and Y-axes show the scores of PC1 and PC2, respectively. (b) The X and Y-axes show the scores of PC1 and PC3, respectively. The size of the points represents the predicted residual error in the prediction by GBLUP for each cultivar.
